# Solvent-Controlled Product Branching in Riboflavin Uncovered by Time-Resolved Infrared and Transient Absorption Spectroscopy

**DOI:** 10.1021/acs.jpclett.6c02005

**Published:** 2026-08-27

**Authors:** Meiyue Liu, Juno Underhill, Jacob F. Jones, Aditya G. Rao, Caleb J. C. Jordan, Somnath Kashid, James A. Smith, J. L. Ross Anderson, Adrian J. Mulholland, Basile F. E. Curchod, Thomas A. A. Oliver

**Affiliations:** † School of Chemistry, 1980 University of Bristol, Cantock’s Close, Bristol BS8 1TS, U.K.; ‡ School of Biochemistry, University of Bristol, University Walk, Bristol BS8 1TD, U.K.

## Abstract

Riboflavin (vitamin B_2_) is an important visible chromophore in flavoproteins and a popular photosensitizer in organic photocatalysis. Despite its importance, a complete understanding of riboflavin’s full photocycle remains undetermined, specifically the factors that govern the relative yields of its singlet and triplet excited states—both playing important roles in electron transfer chemistry. A combination of transient absorption spectroscopy and time-resolved infrared spectroscopy is used to address this problem, tracking the photoexcited states of riboflavin over 9 decades of time under the same experimental conditions. These studies reveal that the triplet quantum yield increases by a factor of 2.7 by merely changing the solvent from DMSO to H_2_O. Electronic structure calculations show that this enhancement is not likely to originate from a change in the associated singlet and triplet state potential surfaces. Instead, we discern solvent-dependent branching, with more efficient internal conversion in DMSO, likely mediated by its stronger hydrogen bonding acceptor character than water. This strong modulation shows that the triplet branching, and thus the reservoir of long-lived states required for electron transfer reactions, can be carefully tuned by the immediate environment of the flavin chromophore.

## Graphical abstract

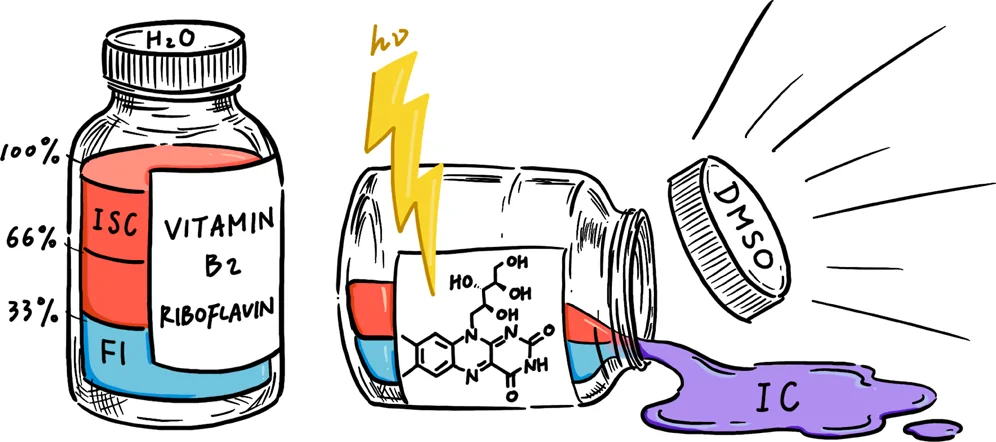

Riboflavin, more commonly known as vitamin B_2_, is the archetypal flavin molecule. As the precursor to flavin mononucleotide (FMN) and flavin adenine dinucleotide (FAD), it underpins metabolic electron transfer reactions and contributes to processes that mitigate oxidative stress.^1–5^ The aromatic isoalloxazine core is the visible chromophore of riboflavin (see chemical structure in Figure 1(a)) and has a significant absorption cross-section in the visible, peaking at ∼450 nm (Figure 1(b,c)). The extended π-electron heteroaromatic structure of riboflavin can stabilize large changes in electron density and accommodate shifts in protonation, giving rise to four readily accessible redox states.^6–8^ Resultantly, flavins are common photoreceptors in LOV (light, oxygen, and voltage)-sensitive domains of proteins, transducing absorbed light into conformational changes that regulate downstream signaling.^9–11^ Their long-wavelength absorption spans a large portion of the blue part of the visible spectrum, enabling their photoreception function in many natural cryptochromes^12–20^ and Blue Light Using FAD (BLUF) domain^21–30^ proteins. Combined, these properties make flavins versatile visible chromophores^5,14,20^ and redox mediators,^4,6,7,31^ and thus popular photosensitizers or photocatalysts in photoinduced electron transfer reactions.^15–17,26–30,32–38^

**Figure 1:**
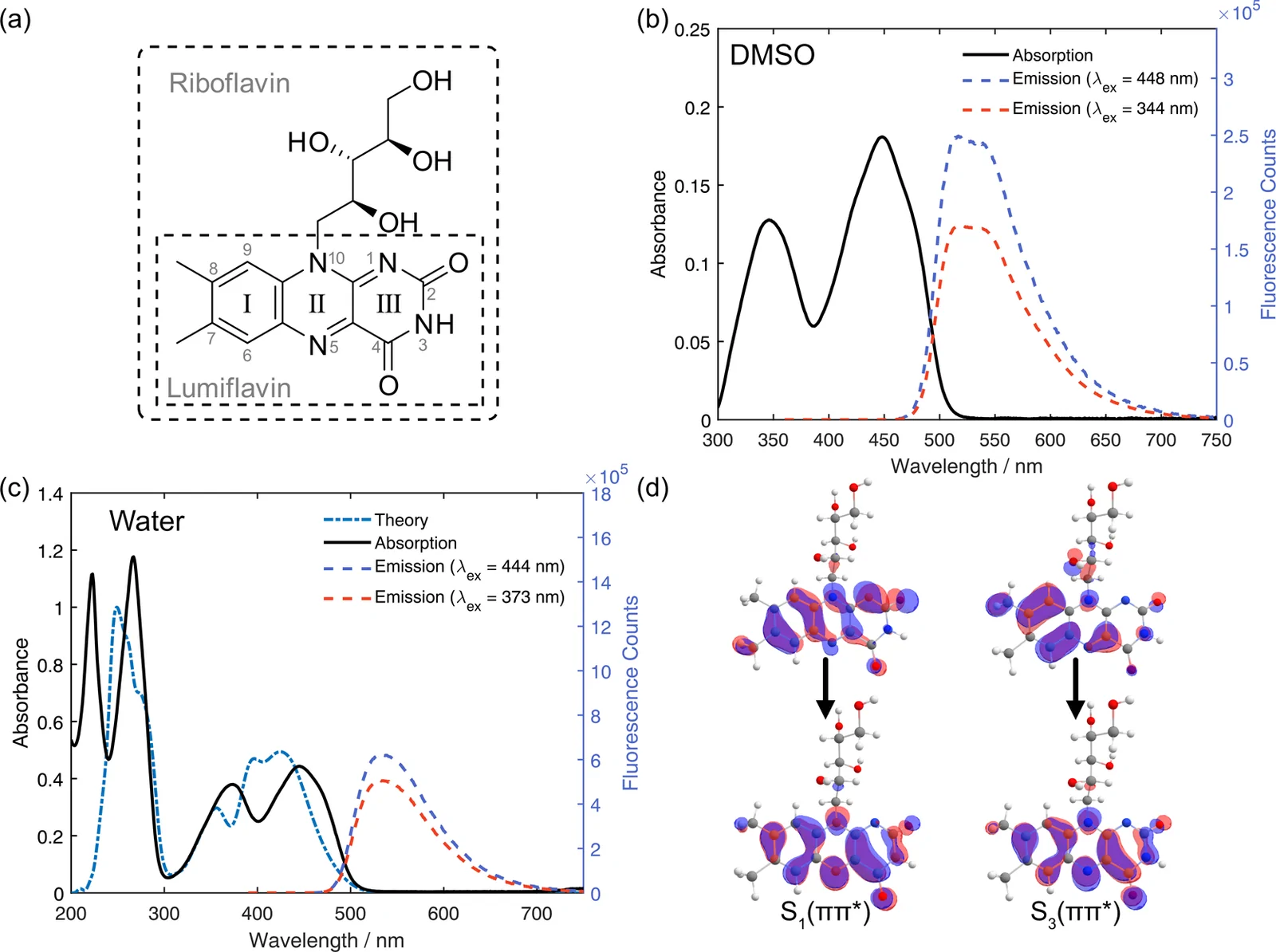
(a) Molecular structures of riboflavin and lumiflavin. The isoalloxazine rings are labeled I, II, and III for ease of reference. (b) Experimental absorption and fluorescence spectra of riboflavin in DMSO. (c) Comparison between the experimental absorption spectrum of riboflavin in water and QM/MM calculations (riboflavin within a shell of 150 QM water molecules) at the LR-TDDFT/TDA/ωB97X-D3/def2-SVP level of theory (broadened with a Gaussian function using a fwhm = 0.165 eV); the latter is normalized to λ = 222 nm. The experimental riboflavin fluorescence spectrum in water is also shown for completeness. The calculated LR-TDDFT/TDA spectrum was shifted by the difference between the LR-TDDFT/TDA and RI-CC2 calculations (0.6 eV). (d) Natural transition orbitals for the two lowest energy π* ← π transitions of riboflavin from a sample snapshot in the QM/MM trajectory (S_1_ and S_3_ states in the Franck–Condon region, respectively), where the arrows connect the hole and particle NTOs associated with each electronic excitation.

Despite the significance of riboflavin, previous time-resolved studies of flavins have either focused on ‘early-’ (femtoseconds–nanoseconds)^39–46^ or ‘late-’time (microseconds and longer)^31–33,47–53^ kinetics, but none have reported on the *complete* photoinduced relaxation cycle of riboflavin in solution. Therefore, key information about the interconversion between singlet and triplet states is missing, e.g., the associated intersystem crossing rate constant or associated quantum yield—values that remain disputed in water.^54^ As both singlet and triplet states can play significant roles in riboflavin electron-transfer reactions, it is important that these key photophysical parameters are rigorously established. To bridge the many decades of time required to follow the riboflavin photoexcited state evolution, we apply transient absorption (TA) and time-resolved infrared (TRIR) ultrafast spectroscopic experiments capable of seamlessly following photoinduced evolution between 150 fs and 0.1 ms under the same experimental conditions. We chose to contrast the photophysics of riboflavin in water, for biological relevance, and in DMSO because its aprotic nature and high dielectric constant allow the molecule to adopt the same neutral oxidized form as in H_2_O,^55^ and the two solvents have different hydrogen-bonding properties. The experimental photophysical findings are supported by quantum chemical calculations. Combined, our studies demonstrate that small changes in the solvent environment lead to a ∼2.7× variation in the triplet quantum yield, despite only small changes to the intersystem crossing rate. The change in intersystem quantum yield is instead underpinned by the fine balance between multiple competing nonradiative decay pathways, thereby demonstrating that the environment can strongly modulate the photochemical product branching in riboflavin.

The experimental absorption and fluorescence spectra of riboflavin in water and DMSO are similar (Figure 1(b,c)), exhibiting two main absorption bands centered at ∼450 and ∼350 nm. The calculated absorption spectrum of riboflavin, from quantum mechanical/molecular mechanics (QM/MM) calculations, reproduces the main features in the experimental absorption spectrum (Figure 1(c)). These are dominated by two long-wavelength maxima at 355 nm (3.49 eV) and 430 nm (2.88 eV), associated with π* ← π transitions, S_3_ and S_1_ states, respectively, in the Franck–Condon region. QM/MM calculations also show two ^1^ *n*π* states within this long-wavelength range (the full stick spectra are given in Figure S15). The two lowest energy ^1^ππ* states are delocalized over the isoalloxazine ring and include only minimal transition density on the ribityl tail (Figure 1(d)). Conversely, the low-lying ^1^ *n*π* states involve promotions from nonbonding orbitals localized on the nitrogen and oxygen atoms to π* orbitals on the isoalloxazine ring, respectively (Figure S12 in the SI).

The photoexcited state decay of riboflavin is explored with TA spectroscopy probing 9 decades of time in both DMSO and water (Figure 2(a) and (c), respectively). The full details of our experiment are given in the SI, with the apparatus drawing inspiration from the LIFEtime experiment at the UK’s Central Laser Facility (STFC).^56,57^ Note that data were acquired with and without dissolved oxygen present, and only the latter is shown in the main text. The negative transients in the TA data at ∼450 nm and ∼555 nm are assigned to ground-state bleach (GSB) and stimulated emission (SE) signals, based on their close correspondence to the respective steady-state spectra. In both solvents, there are also accompanying positive transient features at ∼370, ∼500, and ∼730 nm associated with the riboflavin S_1_ excited-state absorption (ESA).

**Figure 2:**
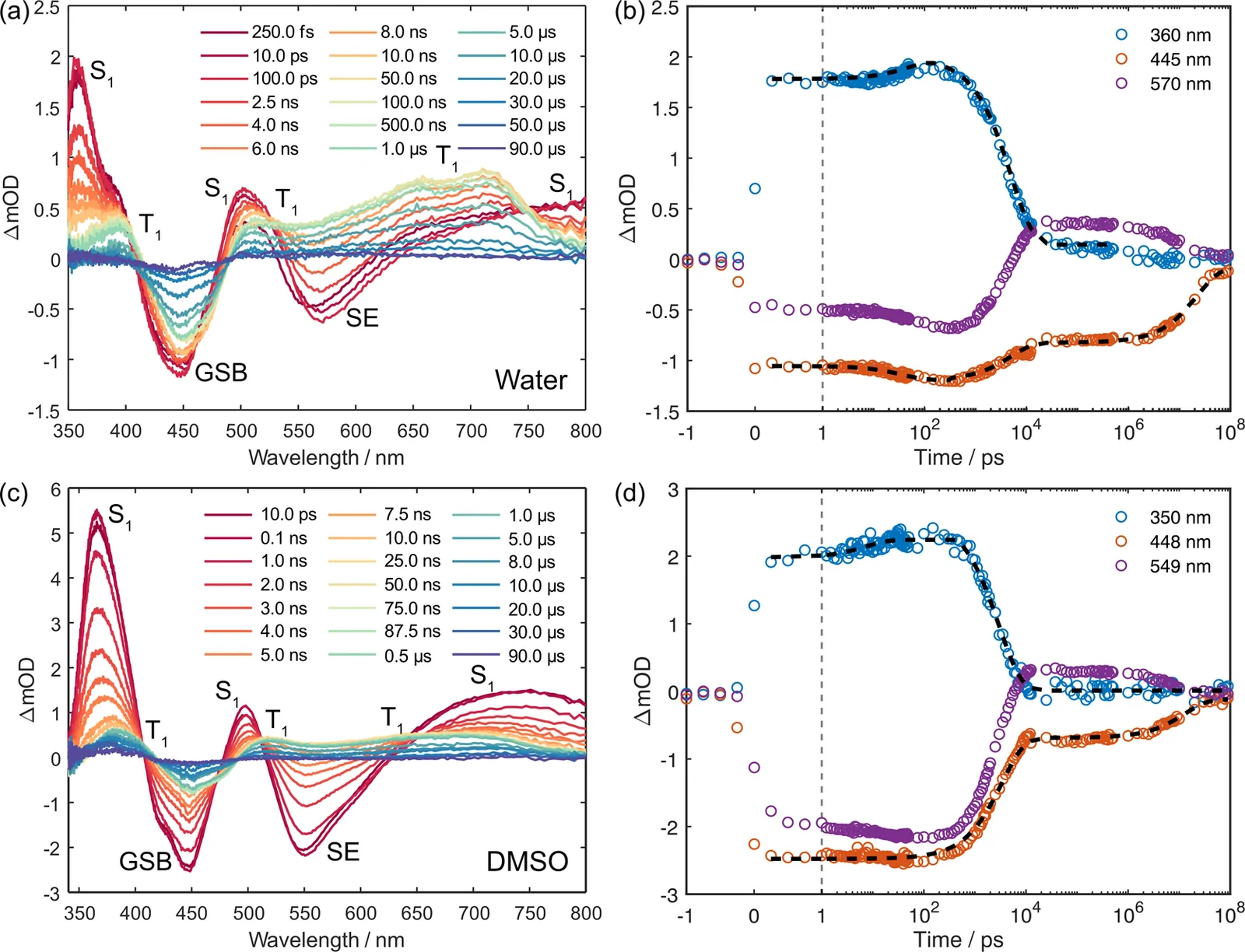
TA spectra at selected time delays for riboflavin in (a) water and (c) DMSO, photoexcited at respective absorption maxima of 445 and 448 nm. Kinetics associated with three representative probe wavelengths for riboflavin are shown in (b) water and (d) DMSO. Overlaid dashed lines are kinetic fits to the data. The data shown are for solutions purged of dissolved oxygen. Data where the dissolved oxygen was not sparged from solutions are shown in Figure S8.

In water (Figure 2(a)), the SE decays on a time scale of several nanoseconds, as expected from the known nanosecond fluorescence lifetime.^39^ On a commensurate time scale, the S_1_ ESA at λ > 470 nm makes a marked spectral evolution and by *t* > 6 ns is replaced by a new triple-peaked positive transient that persists for microseconds. The ESA at λ < 400 nm also evolves on the same time scale, but in a less pronounced way. In DMSO, a similar evolution associated with the S_1_ ESA and SE are observed and, as expected, decay on a nanosecond time scale. The spectral shifting of the positive transient at λ > 500 nm in H_2_O observed on the several-nanosecond time scale is not as significant in the DMSO data (Figure 2(c)). Further, the intensity of the long-lived (microseconds) transient is less pronounced in DMSO than in water, implying a smaller product branching into this species. We assign the microsecond-lived transient to triplet riboflavin, based on the close resemblance to the known triplet absorption spectrum^51^ and the strong oxygen sensitivity observed in its kinetics (Figure S8).

To investigate whether the riboflavin photophysics are altered following excitation to the S_3_ state, instead of the S_1_ state, the pump wavelength dependence of the TA signal was examined. Within our instrument response (145 fs), no additional transient features were observed compared to direct excitation of the S_1_ state (see Figure S5), indicating that the S_3_ → S_1_ internal conversion is very fast, and the subsequent decay kinetics mimic those following direct S_1_ state excitation (Figure S7). Our observation of ultrafast internal conversion is consistent with prior experimental studies that examined only the very earliest time kinetics using an instrument response of 50–60 fs.^43^

Given the clear separation of time scales associated with the observed TA spectral evolution, the photoexcited riboflavin kinetics were extracted by examining the temporal profiles at three different probe wavelengths primarily associated with different species (Figure 2(b) and (d)): (i) 360 nm is dominated by the S_1_ ESA; (ii) the peak of the GSB at λ ∼ 450 nm monitors the return of molecules back to the ground electronic state and contains some contribution from the S_1_ ESA; (iii) ∼550 nm is primarily associated with the SE decay, but at later time delays also overlaps with the triplet absorption.

In water, the S_1_ ESA kinetics at 360 nm are modeled by an exponential rise (50 ± 30 ps) followed by an exponential decay (5.0 ± 0.1 ns). The rise is likely due to vibrational cooling, nuclear reconfiguration in the riboflavin S_1_ state, or potentially a change in oscillator strength upon excited-state evolution; the latter has been suggested previously.^41^ Our theoretical calculations of lumiflavin (structure shown in Figure 1(a)), which has been shown to be an excellent model system for riboflavin (truncating the ribityl tail at the N10 position),^58–60^ reveal that the S_1_ state minimum is stabilized by ∼0.3 eV relative to the Franck–Condon S_1_ geometry (Figure S17), indicating there is appreciable nuclear reorganization on the S_1_ state . The nanosecond decay time constant is in excellent agreement with the reported fluorescence lifetime in water (τ_F_ = 5.1 ± 0.1 ns).^39^ Fits to this probe wavelength exclude the very small amplitude of the overlapping triplet that contributes at longer time delays.

The GSB recovery (kinetics taken at the bleach maximum, λ = 445 nm) is well described by exponential rise (37 ± 7 ps) followed by biexponential decay (5.0 ± 0.1 ns and 23 ± 1 μs). The relative amplitude of the biphasic bleach recovery is directly proportional to the triplet quantum yield (Φ_T_), as the triplet state decay is the sole contributor to the microsecond phase of the bleach recovery (see SI eqs S2–S4). The associated fits to data return normalized pre-exponential factors of 0.33 and 0.67 for the nanosecond and microsecond decay components. The latter amplitude is thus associated with the triplet quantum yield (using eq S3). Our results align with the upper limit of the wide range of triplet quantum yields previously reported for riboflavin in water (0.38–0.7).^48,49,54,61^

Similar probe wavelengths were chosen to extract the kinetic behavior of riboflavin in DMSO, with the exact wavelengths adjusted to account for solvent-induced shifts in the transient maxima. The S_1_ ESA (350 nm) rises with a 7 ± 2 ps time constant and subsequently decays with τ = 3.0 ± 0.1 ns. The GSB (448 nm) recovers biexponentially with time constants of 3.43 ± 0.08 ns and 13 ± 1 μs (Figure 2(d)). The nanosecond ESA decay and GSB recovery are comparable with the literature fluorescence lifetime (3.2 ± 0.1 ns) in DMSO.^39^ A global analysis yielded very similar time constants in both water and DMSO (Figures S9 and S10), demonstrating the validity of the kinetic analysis performed at selected probe wavelengths and confirming that the triplet absorption rises with the decay of the singlet ESA.

In DMSO, the normalized pre-exponential factors associated with the biexponential GSB recovery are 0.76 and 0.24, for nanosecond and microsecond components, respectively. However, we note that the amplitude of the microsecond bleach recovery can be better estimated when the bleach is fully spectrally separated from any other overlapping feature. Thus, in our TA data, the initial bleach intensity at *t* = 0 fs will be underestimated due to the overlapping strong oppositely signed S_1_ ESA, despite being largely unshaped by overlapping ESAs (Figure S4).

A previous TRIR study of riboflavin in DMSO showed that the mid-infrared fingerprint region contains multiple spectrally isolated ground-state bleach vibrations.^40^ This prior study only measured to *t* = 800 ps and was thus unable to capture the intersystem crossing kinetics. Here we use TRIR spectroscopy with the same laser platform as used for TA experiments and can thus examine the multiple phases of bleach recovery and provide a secondary measure of the triplet branching. Our data span 1380–1720 cm^–1^ composed from two overlapping probe windows (Figure 3(a,b)) in DMSO-*d*_6_ and DMSO. The vibrational band assignments of riboflavin have been previously determined and are thus not examined here.^40^

**Figure 3:**
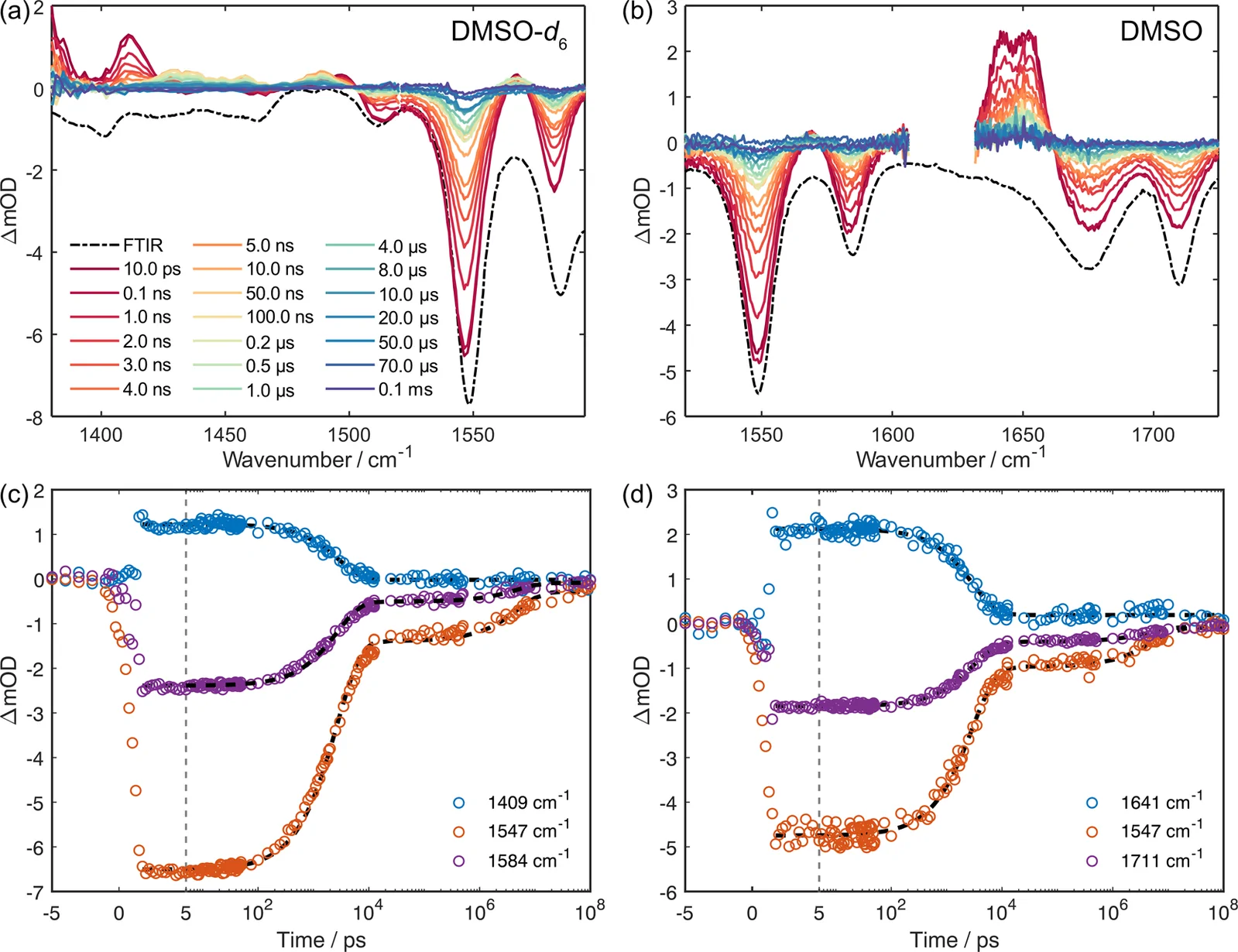
TRIR spectra of riboflavin for the displayed time delays in (a) DMSO-*d*_6_ and (b) DMSO. The respective solvent-subtracted FTIR spectra (inverted) are also overlaid for reference. Kinetic traces at three different probe wavenumbers are displayed for riboflavin dissolved in (c) DMSO-*d*_6_ and (d) DMSO.

The two dominant and isolated bleaches in TRIR data at 1547 and 1711 cm^–1^ (Figure 3(b)) in DMSO were simultaneously fitted to biexponential decay functions, yielding lifetimes of 3.04 ± 0.07 ns and 5.0 ± 0.6 μs (fit to data overlaid in Figure 3(d)). The associated normalized pre-exponential factors are 0.81 and 0.19 for nanosecond and microsecond decay components. The ratio is slightly higher (by 5%) compared to those extracted from the TA data, for the reasons highlighted above.

We note that TRIR data recorded in fully deuterated DMSO yield a shorter riboflavin excited-state lifetime of 2.66 ± 0.03 ns (Figure 3(c)) compared to regular DMSO; however, the ratio of pre-exponential factors remains similar upon solvent deuteration (0.81 and 0.19 in DMSO and 0.82 and 0.18 in DMSO-*d*_6_). A possible explanation for this difference in riboflavin S_1_ lifetime is given in the SI and echoes prior work by Maillard et al.^62^ and Kubitz et al.^63^ TRIR data in H_2_O/D_2_O were not acquired due to the order of magnitude lower riboflavin solubility in water and very strong mid-IR solvent absorption. Due to the lack of the comparator TRIR data, our determination of the triplet quantum yield relies .

Comparing the ISC branching in water and DMSO, less than one-quarter of the excited singlet riboflavin molecules intersystem cross to triplet riboflavin (Φ_T_ = 0.24) in the latter solvent, contrasting with our far higher estimated triplet quantum yield of 0.66 for riboflavin in water. This difference in triplet branching is surprisingly not reflected in the respective fluorescence quantum yields (Φ_F_) of 0.24 ± 0.02 and 0.28 ± 0.04 in DMSO and water, respectively, and in good agreement with prior reports.^39^ Using the combination of experimentally directly determined (τ_F_ and Φ_F_) and derived (Φ_T_) photophysical quantities, *k*_f_, *k*_ISC_, and *k*_IC_ can be determined for riboflavin in H_2_O and DMSO (summarized in Table 1) using eqs S1, S4 and S5. The relative ratios of the three derived rate constants in the two solvents (*k*_H2O_/*k*_DMSO_) are also given in Table 1, enabling direct comparison of the solvent-dependent rates. The fluorescence rate constant is similar for riboflavin in both solvents and in good agreement with independent estimates from a Strickler–Berg analysis (comparison given in Table S1).^64^ The difference in radiative rate constants cannot entirely be rationalized by the change in solvent refractive index and may instead reflect some solvent-dependent mixing between ^1^ *n*π* and the ^1^ππ* (S_1_) state as previously suggested by Weigel et al.^43^ Changes in hydrogen bonding will modulate the energy of the ^1^ *n*π* state relative to the ^1^ππ* state, thereby altering the level of mixing. The net result is to change the oscillator strength of the transition, with greater *n*π* state (optically dark) character leading to a decrease in the radiative rate constant. Conversely, the nonradiative rate constants, *k*_IC_ and *k*_ISC_, vary, with intersystem crossing dominating nonradiative decay in water, but internal conversion for DMSO.

**Table 1:** Summary of Experimentally Determined and Derived Photophysical Quantities for Riboflavin in H_2_O and DMSO^a^

**Experimentally Derived Parameter**	**H**_**2**_ **O**	**DMSO**
Fluorescence lifetime, τ_F_/ns^a^	5.1 ± 0.1	3.2 ± 0.1
S_1_ lifetime/ns^b^	5.0 ± 0.1	3.0 ± 0.1
Fluorescence quantum yield, Φ_F_	0.28 ± 0.04	0.24 ± 0.02
Triplet quantum yield, Φ_T_ ^b^	0.66 ± 0.03	0.24 ± 0.04
Fluorescence rate constant, *k*_F_/s^–1^	(5.6 ± 0.8) × 10^7^	(8.0 ± 0.7) × 10^7^
ISC rate constant, *k*_ISC_/s^–1^	(1.3 ± 0.1) × 10^8^	(8.3 ± 1.4) × 10^7^
IC rate constant, *k*_IC_/s^–1^	(1.0 ± 1.1) × 10^7^	(1.7 ± 0.2) × 10^8^
*k*_F(H2O/DMSO)_	0.7 ± 0.1	
*k*_ISC(H2O/DMSO)_	1.6 ± 0.2	
*k*_IC(H2O/DMSO)_	0.06 ± 0.07	

a Unless otherwise stated, the values given were determined in this study.

b From ref 39.

c Determined from TA measurements, and these time constants were used to calculate the associated rate constants.

Quantum chemical calculations on lumiflavin were used to investigate the molecular origin of the experimentally derived intersystem crossing rate constants in riboflavin. The S_0_ and S_1_ minimum energy geometries of lumiflavin were obtained using DFT/ωB97X-D3/def2-SVP and LR-TDDFT/TDA/ωB97X-D3/def2-SVP, respectively, including an implicit conductor-like polarizable continuum solvent model (CPCM) for both H_2_O and DMSO. A geodesic interpolation pathway^67^ was then constructed to connect the S_0_ and S_1_ state minimum energy structures, and electronic energies were obtained with RI-CC2/def2-TZVP^66^ for S_0_ and S_1_, as well as T_1_, T_2_, and T_3_ states in H_2_O and DMSO (see the SI for full details).

The interpolation pathways shown in Figure 4 indicate that the electronic energies of riboflavin are similar in H_2_O and DMSO in the vicinity of the S_1_ (ππ*) minimum. The triplet state with ^3^ *n*π* character, T_3_ in the present case, which would allow a nonzero spin–orbit coupling with the S_1_ state (ππ*), is substantially higher in energy for both solvents. In the gas phase, the ^3^ *n*π* state is lower in energy and nearly degenerate with the S_1_ (ππ*) state in the Franck–Condon region (see Figure S18). These observations are reminiscent of the earlier work by Salzmann et al. on three flavin-related compounds, highlighting that the ^3^ *n*π* state is destabilized in an aqueous environment.^68^ Our calculations confirm that a similar behavior can be expected for riboflavin in DMSO, rationalizing the similar ISC rate constant determined from experiments (see Table 1). Salzmann et al. explained the nonzero intersystem crossing for flavin in aqueous solution by coupling between S_1_ (ππ*) and a low-lying ^3^ππ* state (in the present case, the T_2_ state), mediated by spin–orbit vibronic coupling. Their intersystem crossing rates for flavin in aqueous solution obtained with DFT/MRCI were on the order of 10^7^–10^8^ s^–1^, in close agreement with the experimentally determined rate constants in the present study for H_2_O and DMSO.

**Figure 4:**
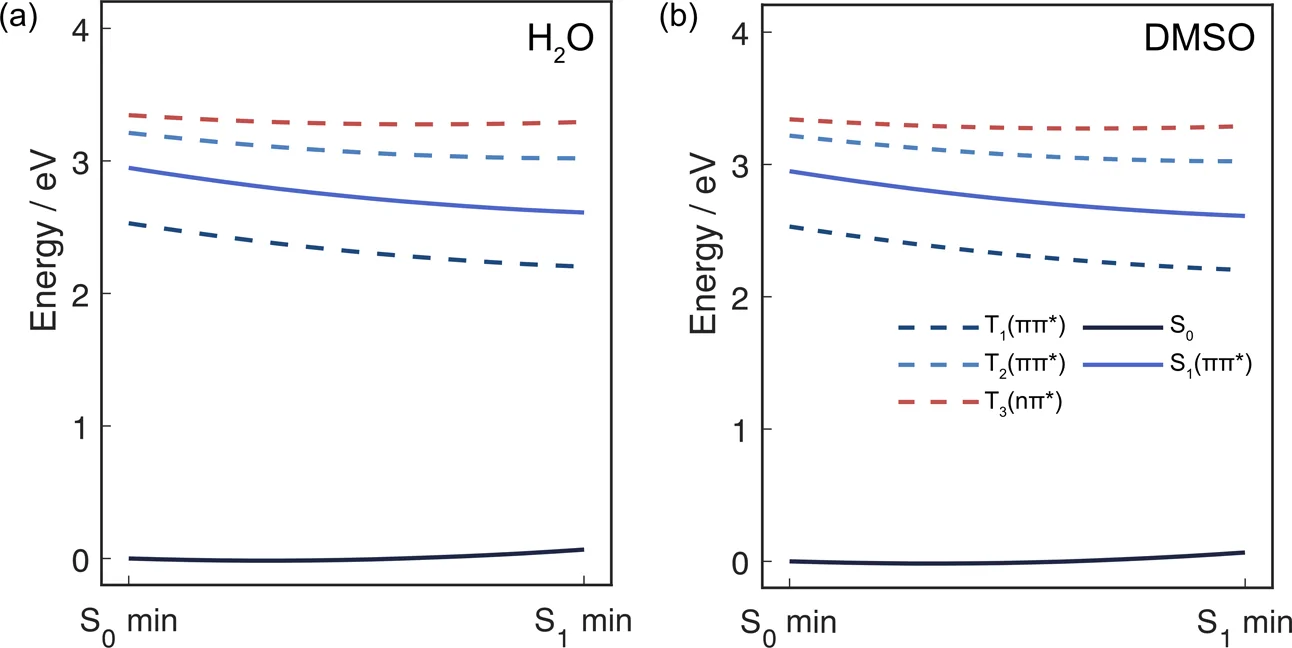
Geodesic interpolation pathways between the S_0_ and S_1_ (ππ*) lumiflavin minima in (a) H_2_O and in (b) DMSO. Electronic energies for the singlet and triplet states presented were obtained with RI-CC2/def2-TZVP using COSMO for implicit solvation, based on optimized geometries obtained with the (LR-TD)­DFT/TDA/ωB97X-D3/def2-SVP/CPCM level of theory (see computational details in SI).

As the experimental analysis in Table 1 indicates, a nonradiative S_1_ → S_0_ pathway must be highly competitive with intersystem crossing and thus further modulates the triplet quantum yield. Electronic structure calculations indicate that it is not likely to originate from changes in the underlying potential energy surfaces (see Figures S16 and S17). Considering the hydrogen-bonding properties of the two solvents, water can act as both a hydrogen-bond donor and acceptor (as illustrated by analyzing our QM/MM output for riboflavin in water, see Figure S13 and Table S2), whereas DMSO acts only as a hydrogen-bond acceptor. Notably, DMSO has a hydrogen-bond acceptor strength more than twice that of water, and water is an equally strong hydrogen-bond donor and acceptor, placing the two solvents at contrasting extremes of hydrogen-bonding character.^69^ Thus, we speculate that H-bonding between the flavin N3–H group (on isoalloxazine ring III) and DMSO solvent molecules somehow mediates internal conversion to S_0_. As water is a weaker H-bond acceptor, this pathway is not as favored and is almost completely turned off (as evident by the close-to-unity sum of fluorescence and triplet yields), giving rise to the significant contrast in internal conversion rates, *k*_IC(H2O/DMSO)_ = 0.06 ± 0.07, and thus altered triplet yield.

The excited-state photochemistry of riboflavin (vitamin B_2_) in DMSO and water was studied with a combination of TA and TRIR spectroscopy, tracking kinetics over nine decades of time, and QM/MM calculations. This approach enabled us to determine the entire photocycle for riboflavin in solution and demonstrate that the triplet quantum yield is strongly dependent on the solvent environment and ∼2.7× larger in water than DMSO. We conclude that the main difference in triplet branching, instead, originates due to a competitive S_1_ → S_0_ internal conversion pathway that is likely enhanced by the hydrogen-bonding acceptor nature of DMSO with the riboflavin N3–H imide bond. H_2_O is a weaker hydrogen-bond acceptor, and thus this pathway is less favored in water, which in turn significantly impacts the triplet quantum yield. This remarkable sensitivity to solvent in the important riboflavin molecule likely underpins why it and related flavin derivatives are common cofactors in multiple different proteins but can be tuned by the surrounding environment to serve different photophysical functions.

## Supplementary Material



## Data Availability

The data underlying this study are openly available at Zenedo at doi: 10.5281/zenodo.22001301.

## Notes

Experimental and theoretical methods; Additional TA and TRIR data and analysis; Further computational data and analysis (PDF)

## References

[1] Fraaije M. W., Mattevi A. (2000). Flavoenzymes: diverse
catalysts with recurrent features. Trends Biochem.
Sci..

[2] Massey V. (2000). The Chemical
and Biological Versatility of Riboflavin. Biochem.
Soc. Trans..

[3] Joosten V., van Berkel W. J. H. (2007). Flavoenzymes. Curr. Opin. Chem.
Biol..

[4] Fagan, R. L. ; Palfey, B. A. Flavin-Dependent Enzymes. In Comprehensive Natural Products II, Liu, H.-W. ; Mander, L. , Eds.; Elsevier, 2010; Vol. 7, pp 37–113.

[5] Pimviriyakul, P. ; Chaiyen, P. Overview of flavin-dependent enzymes. In The Enzymes, Chaiyen, P. ; Tamanoi, F. , Eds.; Academic Press, 2020; Vol. 47, pp 1–36. 10.1016/bs.enz.2020.06.006 32951820

[6] Lukacs A., Zhao R.-K., Haigney A., Brust R., Greetham G. M., Towrie M., Tonge P. J., Meech S. R. (2012). Excited
State Structure
and Dynamics of the Neutral and Anionic Flavin Radical Revealed by
Ultrafast Transient Mid-IR to Visible Spectroscopy. J. Phys. Chem. B.

[7] Heelis P. F. (1982). The photophysical
and photochemical properties of flavins (isoalloxazines). Chem. Soc. Rev..

[8] Kar R. K., Miller A.-F., Mroginski M.-A. (2022). Understanding
flavin electronic structure
and spectra. WIREs Comput. Mol. Sci..

[9] Freddolino P. L., Dittrich M., Schulten K. (2006). Dynamic Switching Mechanisms in LOV1
and LOV2 Domains of Plant Phototropins. Biophys.
J..

[10] Alexandre M. T. A., Domratcheva T., Bonetti C., van Wilderen L. J. G. W., van Grondelle R., Groot M.-L., Hellingwerf K. J., Kennis J. T. M. (2009). Primary Reactions of the LOV2 Domain of Phototropin
Studied with Ultrafast Mid-Infrared Spectroscopy and Quantum Chemistry. Biophys. J..

[11] Freddolino P. L., Gardner K. H., Schulten K. (2013). Signaling mechanisms
of LOV domains:
new insights from molecular dynamics studies. Photochem.& Photobiol. Sci..

[12] Brudler R., Hitomi K., Daiyasu H., Toh H., Kucho K.-i., Ishiura M., Kanehisa M., Roberts V. A., Todo T., Tainer J. A., Getzoff E. D. (2003). Identification of
a New Cryptochrome
Class: Structure, Function, and Evolution. Mol.
Cell.

[13] Brautigam C. A., Smith B. S., Ma Z., Palnitkar M., Tomchick D. R., Machius M., Deisenhofer J. (2004). Structure
of the photolyase-like domain of cryptochrome 1 from Arabidopsis thaliana. Proc. Natl. Acad. Sci. USA.

[14] Essen L.-O. (2006). Photolyases
and cryptochromes: common mechanisms of DNA repair and light-driven
signaling?. Curr. Opin. Struct. Biol..

[15] Solov’yov I. A., Domratcheva T., Moughal Shahi A. R., Schulten K. (2012). Decrypting Cryptochrome:
Revealing the Molecular Identity of the Photoactivation Reaction. J. Am. Chem. Soc..

[16] Solov’yov I. A., Domratcheva T., Schulten K. (2014). Separation of photo-induced radical
pair in cryptochrome to a functionally critical distance. Sci. Rep..

[17] Sjulstok E., Lüdemann G., Kubař T., Elstner M., Solov’yov I. A. (2018). Molecular
Insights into Variable Electron Transfer in Amphibian Cryptochrome. Biophys. J..

[18] Lin C., Top D., Manahan C. C., Young M. W., Crane B. R. (2018). Circadian clock
activity of cryptochrome relies on tryptophan-mediated photoreduction. Proc. Natl. Acad. Sci. USA.

[19] Nielsen C., Nørby M. S., Kongsted J., Solov’yov I. A. (2018). Absorption
Spectra of FAD Embedded in Cryptochromes. J.
Phys. Chem. Lett..

[20] Schwinn K., Ferré N., Huix-Rotllant M. (2020). UV-visible absorption spectrum of
FAD and its reduced forms embedded in a cryptochrome protein. Phys. Chem. Chem. Phys..

[21] Anderson S., Dragnea V., Masuda S., Ybe J., Moffat K., Bauer C. (2005). Structure of a Novel Photoreceptor,
the BLUF Domain of AppA from *Rhodobacter sphaeroides*. Biochemistry.

[22] Masuda S., Hasegawa K., Ono T.-a. (2005). Light-Induced
Structural Changes
of Apoprotein and Chromophore in the Sensor of Blue Light Using FAD
(BLUF) Domain of AppA for a Signaling State. Biochemistry.

[23] Losi A., Gärtner W. (2011). Old Chromophores, New Photoactivation Paradigms, Trendy
Applications: Flavins in Blue Light-Sensing Photoreceptors. Photochem. Photobiol..

[24] Iwata T., Nagai T., Ito S., Osoegawa S., Iseki M., Watanabe M., Unno M., Kitagawa S., Kandori H. (2018). Hydrogen Bonding
Environments in the Photocycle Process around the Flavin Chromophore
of the AppA-BLUF domain. J. Am. Chem. Soc..

[25] Sayfutyarova E. R., Goings J. J., Hammes-Schiffer S. (2019). Electron-Coupled Double Proton Transfer
in the Slr1694 BLUF Photoreceptor: A Multireference Electronic Structure
Study. J. Phys. Chem. B.

[26] Udvarhelyi A., Domratcheva T. (2011). Photoreaction
in BLUF Receptors: Proton-coupled Electron
Transfer in the Flavin-Gln-Tyr System. Photochem.
Photobiol..

[27] Goyal P., Hammes-Schiffer S. (2017). Role of active
site conformational changes in photocycle
activation of the AppA BLUF photoreceptor. Proc.
Natl. Acad. Sci. USA.

[28] Goings J. J., Reinhardt C. R., Hammes-Schiffer S. (2018). Propensity for Proton Relay and Electrostatic
Impact of Protein Reorganization in Slr1694 BLUF Photoreceptor. J. Am. Chem. Soc..

[29] Lukacs A., Tonge P. J., Meech S. R. (2022). Photophysics
of the Blue Light Using
Flavin Domain. Acc. Chem. Res..

[30] He Y., Gil A. A., Laptenok S. P., Fatima A., Collado J. T., Iuliano J. N., Woroniecka H. A., Brust R., Sabbah A., Towrie M., Greetham G. M., Sazanovich I. V., French J. B., Lukacs A., Meech S. R., Tonge P. J. (2025). Enhancing
Proton-Coupled Electron Transfer in Blue Light Using FAD Photoreceptor
AppABLUF. J. Am. Chem. Soc..

[31] Heelis P. F., Parsons B. J., Phillips G. O., Swallow A. J. (1986). One-electron oxidation
of flavins. A flash photolysis and pulse radiolysis study. J. Phys. Chem..

[32] Lu C.-Y., Wang W.-F., Lin W.-Z., Han Z.-H., Yao S.-D., Lin N.-Y. (1999). Generation and photosensitization
properties of the
oxidized radical of riboflavin: a laser flash photolysis study. J. Photochem. and Photobiol. B.

[33] Lu C., Han Z., Liu G., Cai X., Chen Y., Yao S. (2001). Photophysical
and photochemical processes of riboflavin (vitamin B2) by means of
the transient absorption spectra in aqueous solution. Sci. China, Ser. B: Chem..

[34] Jung J., Tollin G. (1981). Transient kinetics
of electron-transfer reactions of
flavodoxins. Biochemistry.

[35] Weber P. C., Tollin G. (1985). Electrostatic interactions
during electron transfer
reactions between c-type cytochromes and flavodoxin. J. Biol. Chem..

[36] Liu B., Liu H., Zhong D., Lin C. (2010). Searching for a photocycle of the
cryptochrome photoreceptors. Current Opinion
in Plant Biology.

[37] Buckel W., Thauer R. K. (2018). Flavin-Based Electron Bifurcation,
A New Mechanism
of Biological Energy Coupling. Chem. Rev..

[38] Laptenok S. P., Lukacs A., Brust R., Haigney A., Gil A., Towrie M., Greetham G. M., Tonge P. J., Meech S. R. (2015). Electron
transfer quenching in light adapted and mutant forms of the AppA BLUF
domain. Faraday Discuss..

[39] Drössler P., Holzer W., Penzkofer A., Hegemann P. (2003). Fluoresence quenching
of riboflavin in aqueous solution by methionin and cystein. Chem. Phys..

[40] Wolf M. M. N., Schumann C., Gross R., Domratcheva T., Diller R. (2008). Ultrafast Infrared Spectroscopy of Riboflavin: Dynamics,
Electronic Structure, and Vibrational Mode Analysis. J. Phys. Chem. B.

[41] Weigel A., Dobryakov A. L., Veiga M., Pérez Lustres J. L. (2008). Photoinduced
Processes in Riboflavin: Superposition of ππ*–nπ*
States by Vibronic Coupling, Transfer of Vibrational Coherence, and
Population Dynamics under Solvent Control. J.
Phys. Chem. A.

[42] Kao Y.-T., Saxena C., He T.-F., Guo L., Wang L., Sancar A., Zhong D. (2008). Ultrafast Dynamics
of Flavins in
Five Redox States. J. Am. Chem. Soc..

[43] Weigel A., Dobryakov A., Klaumünzer B., Sajadi M., Saalfrank P., Ernsting N. P. (2011). Femtosecond Stimulated Raman Spectroscopy of Flavin
after Optical Excitation. J. Phys. Chem. B.

[44] Klaumünzer B., Kröner D., Lischka H., Saalfrank P. (2012). Non-adiabatic
excited state dynamics of riboflavin after photoexcitation. Phys. Chem. Chem. Phys..

[45] Andrikopoulos P. C., Liu Y., Picchiotti A., Lenngren N., Kloz M., Chaudhari A. S., Precek M., Rebarz M., Andreasson J., Hajdu J., Schneider B., Fuertes G. (2020). Femtosecond-to-nanosecond
dynamics of flavin mononucleotide monitored by stimulated Raman spectroscopy
and simulations. Phys. Chem. Chem. Phys..

[46] Xu Y., Peschel M. T., Jänchen M., Foja R., Storch G., Thyrhaug E., de Vivie-Riedle R., Hauer J. (2024). Determining Excited-State
Absorption Properties of a Quinoid Flavin by Polarization-Resolved
Transient Spectroscopy. J. Phys. Chem. A.

[47] Sun M., Moore T. A., Song P.-S. (1972). Molecular luminescence studies of
flavines. I. Excited states of flavines. J.
Am. Chem. Soc..

[48] Grodowski M. S., Veyret B., Weiss K. (1977). Photochemistry
of Flavins. II. Photophysical
Properties of Alloxazines and Isoalloxazines. Photochem. Photobiol..

[49] Moore W. M., McDaniels J. C., Hen J. A. (1977). The Photochemistry of Riboflavin
VI. The Photophysical Properties of Isoalloxazines. Photochem. Photobiol..

[50] Salzmann S., Martinez-Junza V., Zorn B., Braslavsky S. E., Mansurova M., Marian C. M., Gärtner W. (2009). Photophysical
Properties of Structurally and Electronically Modified Flavin Derivatives
Determined by Spectroscopy and Theoretical Calculations. J. Phys. Chem. A.

[51] Bernt
Melø T., Adriana Ionescu M., Haggquist G. W., Razi Naqvi K. (1999). Hydrogen abstraction by triplet flavins. I: time-resolved
multi-channel absorption spectra of flash-irradiated riboflavin solutions
in water. Spectrochim. Acta A Mol. and Bio.
Spectrosc..

[52] Kishore K., Moorthy P. N., Guha S. N. (1991). Pulse radiolysis
study of one electron
oxidation of riboflavin. Int. J. Radiat. Appl.
and Instrum. Part C..

[53] Scholz M., Moučka J., Pšenčík J., Hála J., Dědic R. (2026). Riboflavin – understanding
the dynamics and interactions of the triplet state. Phys. Chem. Chem. Phys..

[54] Görner H. (2007). Oxygen uptake
after electron transfer from amines, amino acids and ascorbic acid
to triplet flavins in air-saturated aqueous solution. J.Photochem. Photobiol. B.

[55] Drössler P., Holzer W., Penzkofer A., Hegemann P. (2002). pH dependence of the
absorption and emission behaviour of riboflavin in aqueous solution. Chem. Phys..

[56] Greetham G. M., Sole D., Clark I. P., Parker A. W., Pollard M. R., Towrie M. (2012). Time-resolved multiple probe spectroscopy. Rev. Sci. Instrum..

[57] Greetham G. M., Donaldson P. M., Nation C., Sazanovich I. V., Clark I. P., Shaw D. J., Parker A. W., Towrie M. (2016). A 100 kHz
Time-Resolved Multiple-Probe Femtosecond to Second Infrared Absorption
Spectrometer. Appl. Spectrosc..

[58] Zanetti-Polzi L., Aschi M., Daidone I., Amadei A. (2017). Theoretical modeling
of the absorption spectrum of aqueous riboflavin. Chem. Phys. Lett..

[59] Wu M., Eriksson L. A. (2010). Absorption Spectra of Riboflavin—A Difficult
Case for Computational Chemistry. J. Phys. Chem.
A.

[60] Climent T., González-Luque R., Merchán M., Serrano-Andrés L. (2006). Theoretical Insight into the Spectroscopy
and Photochemistry of Isoalloxazine, the Flavin Core Ring. J. Phys. Chem. A.

[61] Islam S. D. M., Penzkofer A., Hegemann P. (2003). Quantum yield of triplet formation
of riboflavin in aqueous solution and of flavin mononucleotide bound
to the LOV1 domain of Phot1 from Chlamydomonas reinhardtii. Chem. Phys..

[62] Maillard J., Klehs K., Rumble C., Vauthey E., Heilemann M., Fürstenberg A. (2021). Universal quenching of common fluorescent
probes by
water and alcohols. Chem. Sci..

[63] Kubitz M.
K., Haselbach W., Sretenović D., Bracker M., Kleinschmidt M., Kühnemuth R., Seidel C. A. M., Gilch P., Czekelius C. (2023). Increasing
the Fluorescence Quantum Yield and Lifetime of the Flavin Chromophore
by Rational Design. ChemPhotoChem..

[64] Zirak P., Penzkofer A., Mathes T., Hegemann P. (2009). Photo-dynamics of roseoflavin
and riboflavin in aqueous and organic solvents. Chem. Phys..

[66] Christiansen O., Koch H., Jørgensen P. (1995). The second-order
approximate coupled
cluster singles and doubles model CC2. Chem.
Phys. Lett..

[67] Zhu X., Thompson K. C., Martínez T. J. (2019). Geodesic interpolation for reaction
pathways. J. Chem. Phys..

[68] Salzmann S., Tatchen J., Marian C. M. (2008). The photophysics
of flavins: What
makes the difference between gas phase and aqueous solution?. J. Photochem. Photobiol., A.

[69] Abraham M. H. (1993). Scales
of solute hydrogen-bonding: their construction and application to
physicochemical and biochemical processes. Chem.
Soc. Rev..

