# Supplementary material for: Solvent-Controlled Product Branching in Riboflavin Uncovered by Time-Resolved Infrared and Transient Absorption Spectroscopy
Source: J Phys Chem Lett. 2026 Aug 27;17(38):10887–94. doi: 10.1021/acs.jpclett.6c02005 (PMC13618035; doi:10.1021/acs.jpclett.6c02005)
Supplement: Supplementary file 1 [file jz6c02005_si_001.pdf]

Supplementary Information for:

Solvent-Controlled Product Branching in Riboflavin Uncovered by Time-Resolved Infrared and Transient Absorption Spectroscopy

Meiyue Liu,[1‡] Juno Underhill,[1‡] Jacob F. Jones,[1] Aditya G. Rao,[1] Caleb J. C. Jordan,[1] Somnath Kashid,[1] James A. Smith,[1] J. L. Ross Anderson,[2] Adrian J. Mulholland,[1] Basile F. E. Curchod,[1] and Thomas A. A. Oliver[1]*

[1] School of Chemistry, Cantock's Close, University of Bristol, BS8 1TS, UK

[2] School of Biochemistry, University Walk, University of Bristol, BS8 1TD, UK

[‡] These authors contributed equally to this work

*Author for correspondence: tom.oliver@bristol.ac.uk

S1.1 Experimental Apparatus

Femtosecond-to-microsecond transient absorption (TA) and time-resolved infrared (TRIR) spectroscopy experiments were established using two synchronised amplified Yb:KGW lasers (2× Pharos PH2, Light Conversion) seeded by the same 80 MHz oscillator. This common seeding enables precise time synchronisation of the two amplifiers, and the ability to electronically delay one amplifier relative to another by up to t = 1 ms in multiples of 12.5 ns (corresponding to one oscillator round trip) or 10 μs (corresponding to the native repetition rate of the regenerative amplifier).

The output of the primary amplifier was used to pump a commercial optical parametric amplifier (OPA, Orpheus-HP, Light Conversion), capable of generating tuneable laser excitation pulses between 230 and 2500 nm. 80% of the second amplifier was used to pump a second OPA (Orpheus-One, Light Conversion) to generate near-infrared pulses between 1400 – 2200 nm. These were mixed with a portion of the 1030 nm fundamental, in a difference frequency generation (DFG) stage (LYRA, Light Conversion) to generate the required mid-infrared (IR) probe pulses (4000 – 13500 nm) for TRIR experiments. A small portion of the remaining 20% of the secondary amplifier output was used to generate a white light supercontinuum (WLC) probe for transient absorption spectroscopy measurements. The 1030 nm driving force for the WLC was attenuated with a waveplate-polariser pair (WPH10M-1030 and WP25M-UB1, Thorlabs) and then focused (f = 10 cm) into a continually rastered 5 mm thick, 25 mm diameter CaF_2 plate (Crystran).

Both TA and TRIR experiments share common optical paths on the same laser table (see schematic in Figure S1). Broadband dielectric mirrors (TFMS-25.4C05-2/7, OptoSigma) are used in the pump line to minimise accumulated reflection losses and ensure sufficient pump power at the respective sample position. The output of the pump OPA is divergent and was

corrected for with a 1:1 telescope where the position of the second lens was altered to achieve a fully collimated beam over ~8 m. The pump beam is optically delayed relative to the probe by four reflections on two UV-enhanced aluminium retroreflectors (UBBR2.5-1UV, MKS Newport) mounted on a 1.0 m motorised delay stage (PRO190LM-1000-TT1-E1-CMS1-PL2-TAS, Aerotech). After the delay stage, the pump beam is routed to either TA or TRIR spectrometers and focused into the respective samples using f = 25 cm UV-fused silica lenses (LA4158, Thorlabs).

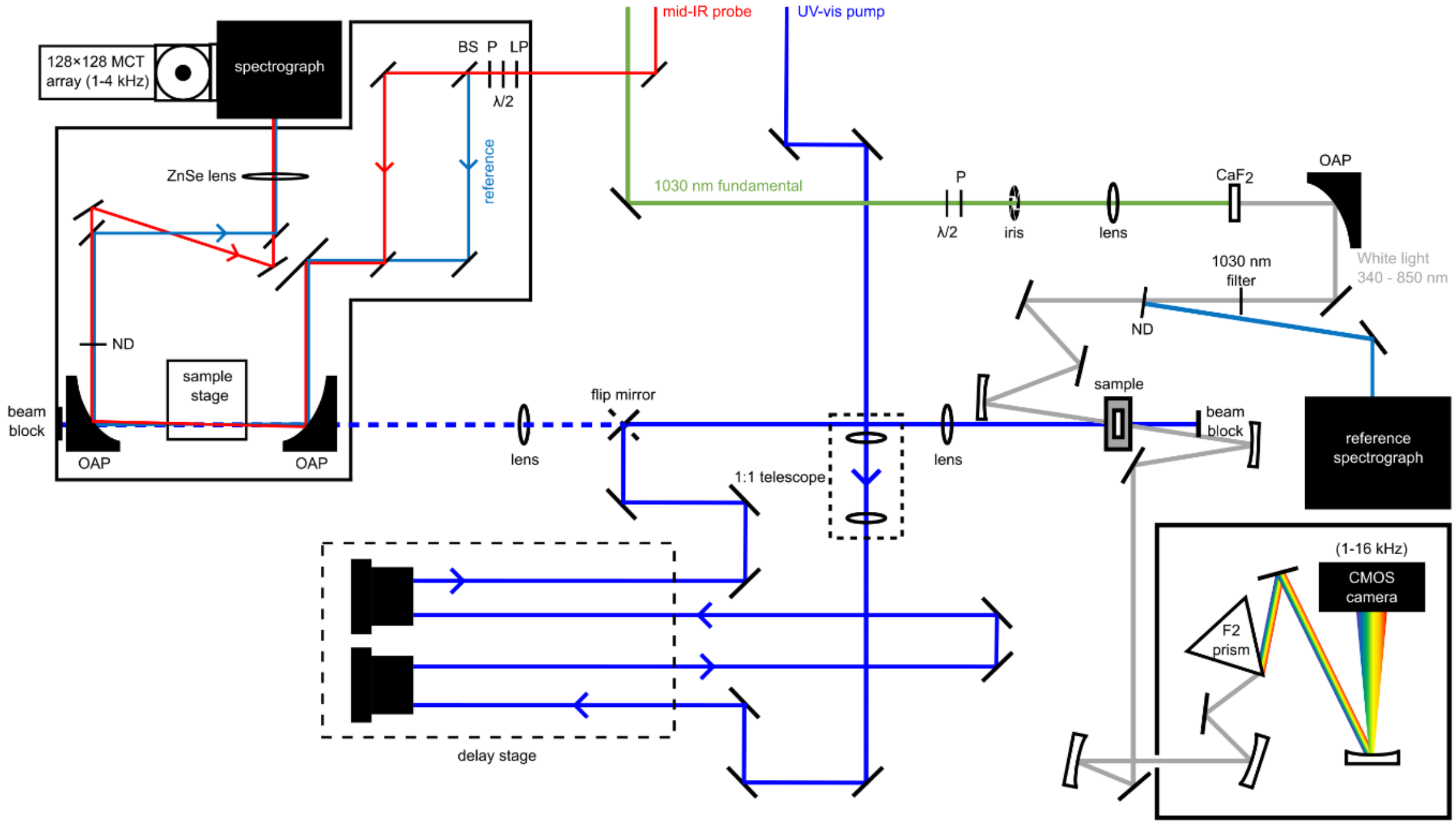

Figure S1. Schematic layout of the optical beam paths associated with TA and TRIR experiments. The mid-IR probe line is comprised from protected gold mirrors. Except for the two UV-enhanced aluminium retroreflectors, broadband dielectric mirrors are used in the pump line to minimise reflection losses. Legend for other optical elements: LP, long-pass filter; λ/2, half waveplate; P, polariser; BS, beam splitter; OAP, off-axis parabolic mirror; ND, neutral density filter.

Protected gold mirrors are used to route the mid-IR probe line from its generation in the LYRA to the mid-IR detector. After the LYRA DFG unit, the mid-IR beam is enclosed in a lens tube and directed to a compact purge box containing the TRIR spectrometer. The LYRA, lens tubes, mid-IR box, and spectrograph are purged by a positive pressure of water-free nitrogen gas to remove water vapour and CO_2 gas. A waveplate-polariser pair (WPLH05M-5300 and WP25H-

Z, Thorlabs) serves the dual purpose of attenuating the mid-IR power at the sample position and setting the magic angle polarisation condition (54.7°). A 80% transmissive, 20% reflective ZnSe beam splitter (Evaporated Optical Coating) separates the mid-IR beam into probe and reference pulses before the sample. The probe and reference beam are focused into the sample and re-collimated by a pair of protected gold 90° off-axis parabolic mirrors (MPD269H-M01, Thorlabs) with a reflective focal length (RFL) of 152.4 mm. The pump beam is focused through a horizontal hole on the first OAP and spatially overlapped at the focus of the mid-IR probe beam in the sample. The mid-IR probe and reference laser beams are focused into a monochromater (SP2150, Princeton Instruments equipped with of 75 grooves/mm and 30 grooves/mm gratings) using a 2-inch diameter ZnSe lens (R5001014634-23040, Edmund Optics) and imaged onto a 128-by-128 2D pixel liquid nitrogen cooled mercury cadmium telluride (MCT) 2D array detector (2DMCT, Phasetech). To avoid detector saturation, the mid-IR probe and signal are attenuated after the sample using neutral density filters (Spectragon). When only 32 rows of the detector are read out, the 2D array detector can operate at a maximum repetition rate of 4 kHz, and allows simultaneous imaging of the probe and reference beams. The reference beam is used to correct for laser intensity fluctuations on a shot-to-shot basis. We implement the shot-to-shot normalisation using a reference correlation matrix,[1-5] that accounts for differences in imaging probe/signal and reference beams through the spectrograph and uncorrelated pixel noise.

The noise characteristics of the TRIR instrument are illustrated in Figure S2, comparing averaged ΔA spectra for a different number of total laser shots in an unpumped sample, with and without reference detection. These data show that increasing the total number of averaged ΔA spectra from 2,500 to 100,000 improves the overall averaged standard deviation (σ) and noise floor. However, due to the inherently high stability of the mid-IR probe generated with the Yb:KGW pumped OPA, the signal-to-noise of the averaged data appears similar either with

or without reference detection, especially after averaging over 100,000 ΔA spectra. However, we note that reference detection does significantly improve the standard deviation.

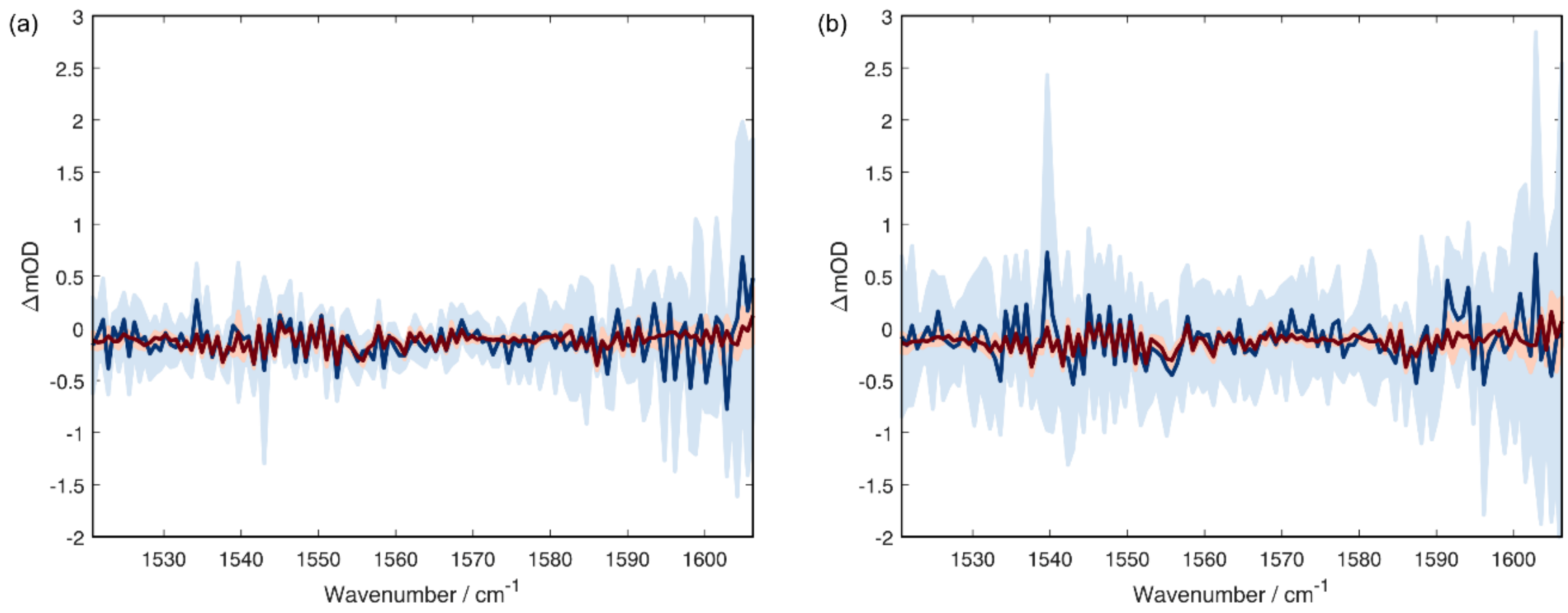

Figure S2. Averaged ΔA spectra (a) with and (b) without reference detection. The blue and red traces are obtained from averaging over 2,500 or 100,000 transient difference spectra. The colour-coded shaded regions correspond to the standard deviation within these different datasets.

In TA experiments, the WLC probe is collimated by a UV-enhanced aluminium off-axis parabolic mirror (MPD249-F01, Thorlabs) with an RFL of 10 cm, and the residual 1030 nm fundamental was removed with a filter (Schott KG3). An OD = 0.2 neutral density filter is used to reflect a small portion of the WLC probe, which is used as a reference beam. The main portion is focused using a *f* = 15 cm UV-enhanced aluminium concave mirror (CM508-150-F01, Thorlabs) into the sample. The WLC probe and co-linear signal are subsequently directed to a home-built F2 prism spectrograph coupled to an FPGA 512-pixel linear array (Glaz LineScan-I-Gen2, Synertronic Designs with S12198-512Q CMOS sensor, Hamamatsu). The standard deviation of the WLC before and after referencing is plotted against the pixel axis (Figure S3) for transient data with absence of a pump pulse. From these data, it is evident there the baseline which should be flat is significantly structured without reference detection, even when averaging over 100,000 delta signals. Referencing improves this significantly, that a similar baseline is achieved by averaging for 5000 ΔA spectra. With reference detection, we achieve a sensitivity of ~ 10 μOD, comparable to previously reported noise characterisations

of white-light continua generated in CaF_2 crystals pumped by Ti:Sapphire lasers where reference detection was utilised.[6, 7]

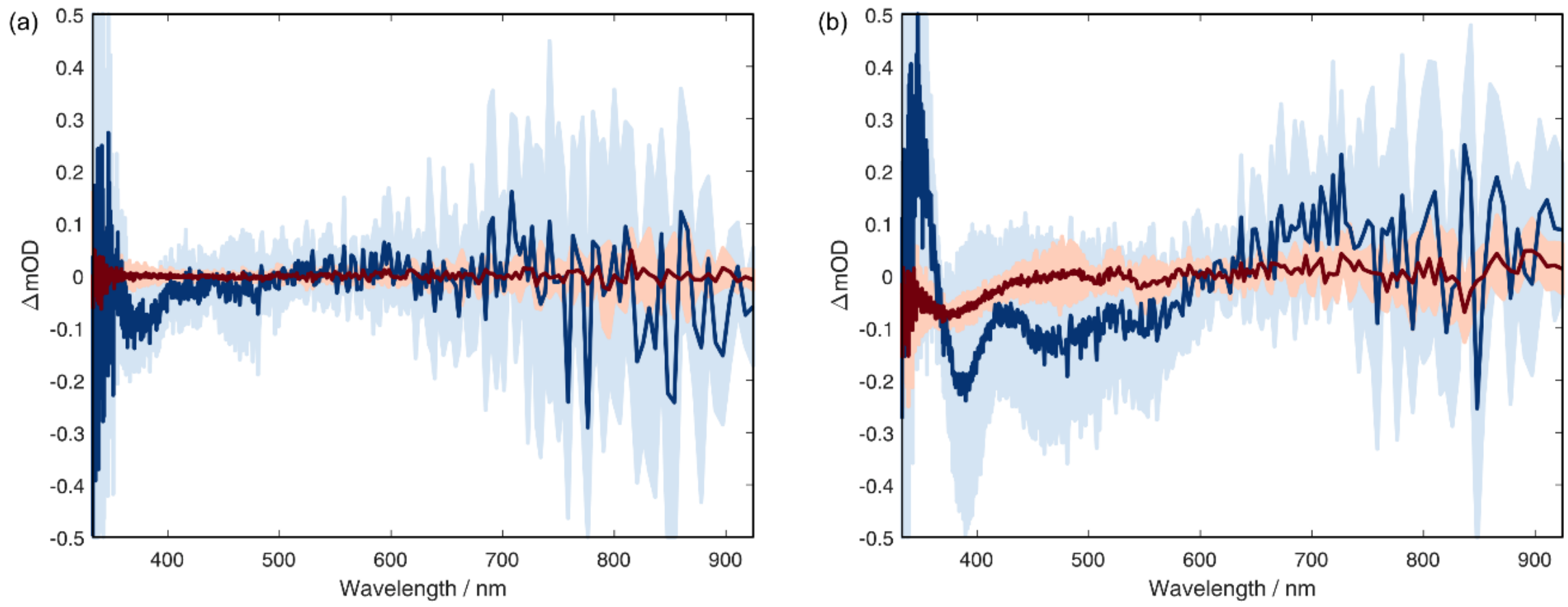

Figure S3. Change of optical density between pump on and pump off with the pump beam blocked (a) with and (b) without reference detection. The blue ΔmOD baseline is obtained from averaging 5,000 ΔA spectra, and the red line 100,000 ΔA data. The shaded blue and red regions correspond to ± σ.

All the data were recorded using home-built control software (LabVIEW, National Instruments), and analysed using customised code (MATLAB, MathWorks).

TA data of riboflavin (RF) were acquired in water and dimethyl sulfoxide (DMSO) solvents, and TRIR data recorded in DMSO and DMSO-d_6. Riboflavin (>99.9%) was purchased from Sigma Aldrich (USP) and used without further purification. For TA experiments, riboflavin was dissolved in water and filtered (0.2 μm PTFE filters, Fisherbrand), yielding solutions with OD of 0.05 at 373 nm and 0.09 at 445 nm in a 200 μm pathlength homebuilt Harrick flow cell. The concentration of these solutions was limited by riboflavin's water solubility. Similarly, riboflavin was dissolved in DMSO and filtered to generate solutions with OD of 0.21 at 448 nm and 0.14 at 344 nm in a 50 μm pathlength Harrick cell. Similar concentrations were used for the TRIR experiments but with an increased pathlength of 200 μm to enhance the mid-infrared absorption (shown in Figure 3 of the main paper). In TA measurements, the pump was focused into the sample with a spot size of 160 μm (diameter), and the power attenuated to 110 nJ/pulse (3.8 GW/cm^2), and the corresponding white light continuum probe spot size was

60 μm. The TA instrument response function (IRF) of 145 fs was determined from the solvent-only response. TRIR experiment used a larger spot size of 420 μm and a correspondingly higher excitation energy of 625 nJ/pulse (3.1 GW/cm^2), and a larger mid-IR probe spot size of 200 μm. This ensures that similar power densities are used in both experiments.

The solutions were sparged of dissolved oxygen by bubbling solutions for 45 minutes with nitrogen gas prior to measurements. To ensure that solutions remained oxygen-free throughout transient measurements, a positive pressure of N_2 filled the headspace above the solution.

The synchronised dual-Pharos amplifier laser system enables flexible pulse picking and thus facile selection of desired operating repetition rates. For the experiments detailed in this study, a 2 kHz pump and 4 kHz probe scheme was chosen, mainly limited by the maximum readout of the 2D MCT detection system. Although the CMOS detector can operate at a higher repetition rate (~16 kHz), the same repetition rates were used for both experiments to allow direct comparison.

The steady-state FTIR and absorption spectra were acquired with the IRTracer-100 FTIR (Shimadzu) and Cary 60 UV-vis (Agilent) spectrometers, respectively. The steady-state fluorescence spectra were recorded with a commercial fluorimeter (FS5, Edinburgh Instruments), and the fluorescence quantum yields were determined using an integrating sphere module (SC-30) attached to the same fluorimeter. The DMSO solvent fluorescence background was subtracted from riboflavin measurements acquired in that solvent.

S1.2 Quantum Yield Calculations

For riboflavin, the triplet state is generated exclusively *via* ISC from the S_1 state, *e.g.* there are no multi-body pathways such as singlet fission in operation. Further, the triplet state decays only to S_0, with no repopulation of the S_1 state *via* thermally activated delayed fluorescence or triplet-triplet annihilation processes. Under these conditions, the triplet quantum yield (Φ_T) is equivalent to the intersystem crossing quantum yield (Φ_{ISC}).

From the measured fluorescence quantum yield (Φ_F) and experimentally determined fluorescence lifetime (τ_F), the fluorescence rate constant (k_F) can be obtained through the well-known relation:

$$k_F = \frac{\Phi_F}{\tau_F} \qquad \text{(S1)}$$

The Φ_T can be estimated from the relative amplitude of the ground state bleach recovery associated with microsecond lifetimes. Where the ground state bleach kinetics take the functional form (excluding the convolution with the Gaussian IRF):

$$-A_1 e^{-t/\tau_1} - A_2 e^{-t/\tau_2} \qquad \text{(S2)}$$

Here A_1 and A_2 are the amplitudes associated with the nanosecond and microsecond bleach recovery time constants (τ_1 and τ_2, respectively).

From this relative ratio, Φ_T is determined thus:

$$\Phi_T = \frac{A_2}{A_1 + A_2} \qquad \text{(S3)}$$

Meaning the rate constant for ISC from the singlet state (k_{ISC}) can be calculated *via*:

$$k_{ISC} = \frac{\Phi_T}{\tau_F} \qquad \text{(S4)}$$

And thus, the rate of internal conversion from the S_1 state can be obtained:

$$k_{\mathrm{IC}} = \frac{1}{\tau_{\mathrm{F}}} - k_{\mathrm{ISC}} - k_{\mathrm{F}} \qquad \text{(S5).}$$

S2. Computational details

S2.1 Absorption spectra of riboflavin from QM/MM

Riboflavin was optimised in the gas phase in the ground electronic state using DFT with the ωB97X-D3 functional[8] and the def2-SVP basis set[9] using TeraChem v1.9-2023.06-dev.[10-12] This geometry was used as a starting point for classical molecular dynamics simulations under periodic boundary conditions using AMBER v24.[13] The structure was initially solvated in a truncated octahedral water box of TIP3P molecules using the solvatebox plugin of Tleap in Ambertools v24.[14] The unit cell parameters were a = b = c = 63.008 Å; α = β = γ = 109.471°. The time step for the simulations was 2 fs, utilising the SHAKE algorithm to constrain hydrogen atoms.[15] The non-bonded cut-off was 12 Å, and the long-range interactions were handled using the Particle Mesh Ewald algorithm.[16] The Langevin thermostat with a collision frequency of 1.0 ps^{-1} was used throughout the simulations.[17] The Berendsen barostat was used to maintain pressure, with a pressure coupling timescale of 1.0 ps.[18] The force field parameters used for riboflavin were calculated using the mdgx tool in AMBER v24[19-21] as described in earlier studies for photoactive biomolecules.[22] Briefly, the approach involved fragmentation of riboflavin, conformation generation around the C-N10 dihedral (connecting the isoalloxazine ring to the ribityl tail), followed by charge density calculations at the HF/6-31G* level of theory using ORCA v5.0.3.[23] The final charges were computed using a multi-conformational RESP fitting strategy.[24] The bonded parameters were taken directly from the GAFF2 force field. The quality of the charges and force field parameters was evaluated by comparison of the MM and QM profiles. The system was then minimised in 1000 steps using the steepest-descent algorithm. The minimised system was then heated to 300 K for 1 ns, before being equilibrated for 1 ns in the NPT ensemble (300 K, 1 bar). The system was considered equilibrated when the density reached 0.98 g/cm^3. The production simulations were then carried out for 20 ns under NVT conditions. QM/MM molecular dynamics was then initiated from the last step of the NVT

production simulation.[25] The QM region used was the riboflavin molecule described using DFT (ωB97X-D3, def2-SVP), while the MM region was the surrounding water molecules, described with TIP3P.[26] The interaction between the QM and MM regions was described using electrostatic embedding with a point charge cutoff of 8 Å. 10 ps dynamics were conducted in the NVT ensemble. The system reached 300 K, after 2 ps for the whole system, as well as the composite QM and MM regions. Hydrogen bonding interactions between the riboflavin and water molecules were calculated using cpptraj in Ambertools v24 for each frame within the MM and QM/MM trajectories. 100 snapshots, sampled every 0.08 ps, were extracted between 2 ps and 10 ps of the QM/MM trajectory for further processing.

15 excited states were calculated for each snapshot with linear-response time-dependent density functional theory (LR-TDDFT) at the same level of theory, employing the Tamm-Dancoff approximation (TDA).[27] The surrounding water molecules were then systematically included into the QM region for the excitation energy calculations to probe the effect of the solvent environment on riboflavin. Shells of water molecules around riboflavin of radii in the range 2-6 Å were selected, leading to 0, 7, 37, 71, 101, and 150 water molecules in the QM region. Furthermore, the excitation energies of each snapshot were recalculated in vacuum, thus totalling 7 sets of calculations. The excitation energies and transition dipole moments of each snapshot were broadened with a Gaussian function with a FWHM of 0.165 eV in the same procedure used in Ref. 28 and the broadened spectra is shown in Figure S13.[28]

The natural transition orbitals (NTOs) were calculated using TheoDORE 2.4[29] and visualised in ChemCraft.[28, 30]

S2.2 Interpolated pathways for lumiflavin model

Lumiflavin, a model system for riboflavin (truncated at the ribityl tail at the N10 position), was optimized on the ground electronic state to obtain S_{0min} for the gas phase and two solvents, DMSO and water. This was performed using density functional theory (DFT) at the ωB97X-D3 functional, the def2-SVP level of theory with the conductor-like polarizable continuum model (CPCM) for the solvents, in ORCAv6.0.1.[23] The dielectric constants (epsilon) used for the two solvents, DMSO and water, were 45.0 and 80.0, respectively. 15 excited states were calculated at the same level of theory using linear-response time-dependent density functional theory employing the Tamm-Dancoff approximation (LR-TDDFT/TDA). S_{0min} was then optimized on the S_1 electronic state using LR-TDDFT/TDA at the same level of theory (ωB97X-D3 and def2-SVP) to obtain S_{1min}. Optimisation was conducted in the gas phase to locate the S_{1min} ($n\pi^*$) and S_{1min} ($\pi\pi^*$) minima, and in both solvents to find the S_{1min} ($\pi\pi^*$) minima. Frequency calculations were performed on all optimized geometries to confirm that they were indeed minima on their respective potential energy surfaces. The located S_{0min} and S_{1min} were used to construct a geodesic interpolation pathway composed of 16 structures connecting these two critical structures.[31] Resolution of identity second-order approximate coupled cluster (RI-CC2[32]) calculations were performed for each structure along this path to map the electronic energies of the S_0 and S_1 singlet states and the T_1–T_3 triplet states in both solvents. These energies were calculated using Turbomole v7.8,[33] the COSMO implementation[34] for the solvents, and a def2-TZVP basis set.

The RI-CC2 wave function was analysed in terms of Natural transition orbitals (NTOs) calculated using TheoDORE 2.4.[29] These NTOs were visualised in ChemCraft.[30]

S3. Experimental Results

S3.1 Comparison of Transient Spectra with Steady State Absorption Spectra

As is evident in Figure S4, the peak and the red edge of the GSB remain largely unchanged by the overlapping positive ESA. This comparison motivates examining the bleach recovery kinetics by plotting the peak of the GSB feature.

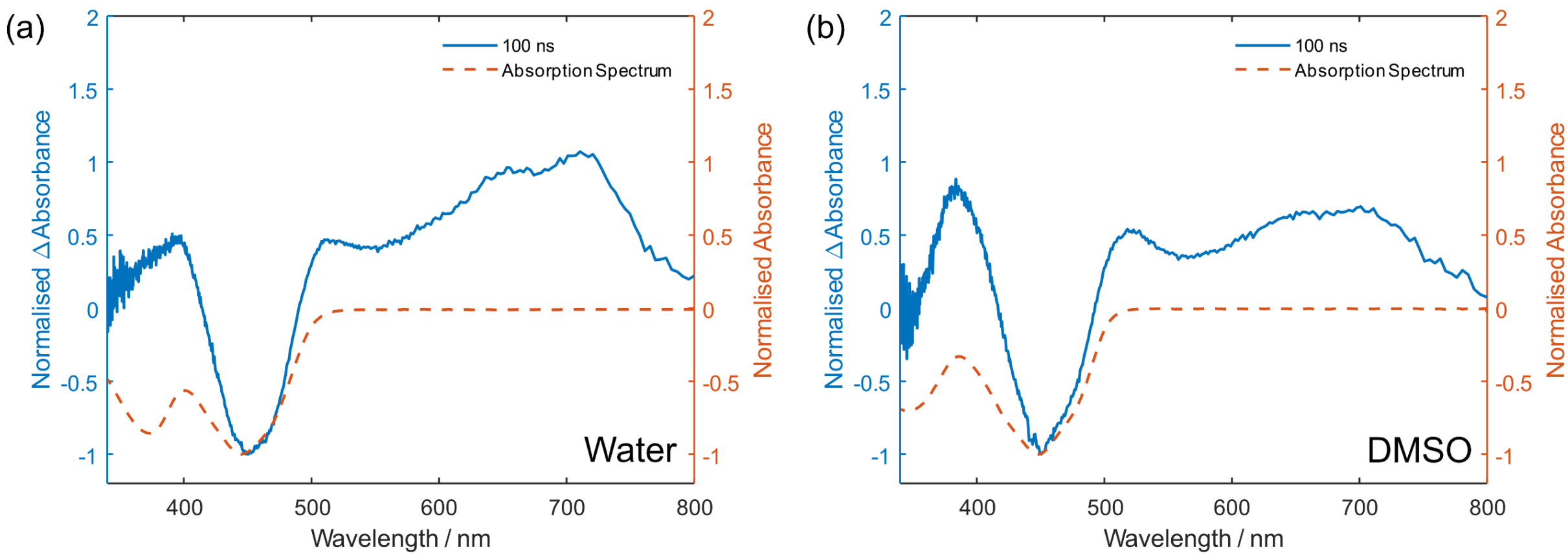

Figure S4. Transient absorption spectrum overlaid with inverted steady-state absorption spectra of riboflavin in (a) water and (b) DMSO.

S3.2 Pump Wavelength Dependent Transient Absorption Measurements

Data acquired at shorter pump wavelengths, exciting the $2^1\pi\pi^*$ state, are shown in Figure S5, with TA data acquired after exciting the $1^1\pi\pi^*$ state shown in Figure 2 (main manuscript). The four sets of TA data are shown as false colour contour plots in Figure S6. No additional transient features are observed when exciting to the second $^1\pi\pi^*$ state. There is a minor difference between the data acquired at different pump wavelengths in the same respective solvent: the rise of the excited state absorption features is more pronounced, presumably as there is more internal vibrational energy that must be dissipated.

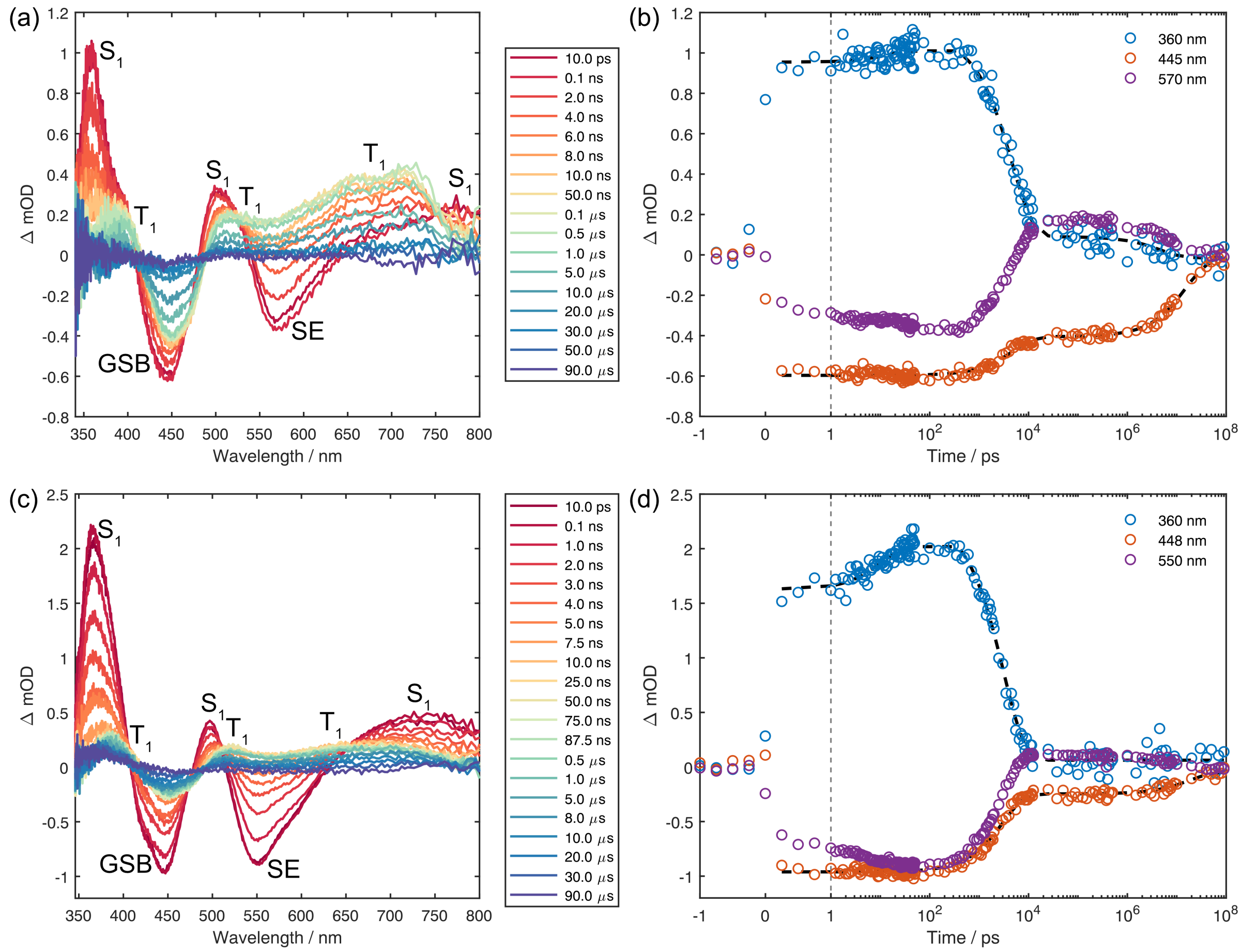

Figure S5. TA data at selected time delays for riboflavin in (a) water and (c) DMSO, using 373 nm and 344 nm pump wavelengths, respectively. Panels (b) and (d) show the kinetics at three probe wavelengths in water and DMSO, respectively. The data presented here are for riboflavin samples where the dissolved oxygen was sparged with N_2 gas.

To assist in the direct comparison, kinetic traces at 360 nm and the respective SE maxima in water and DMSO are normalised and overlaid (Figure S7). Apart from differences in the initial

rise kinetics following excitation to the first or second ππ* states, the intersystem crossing and subsequent ground state bleach recovery (kinetics not shown) display no pump wavelength dependence.

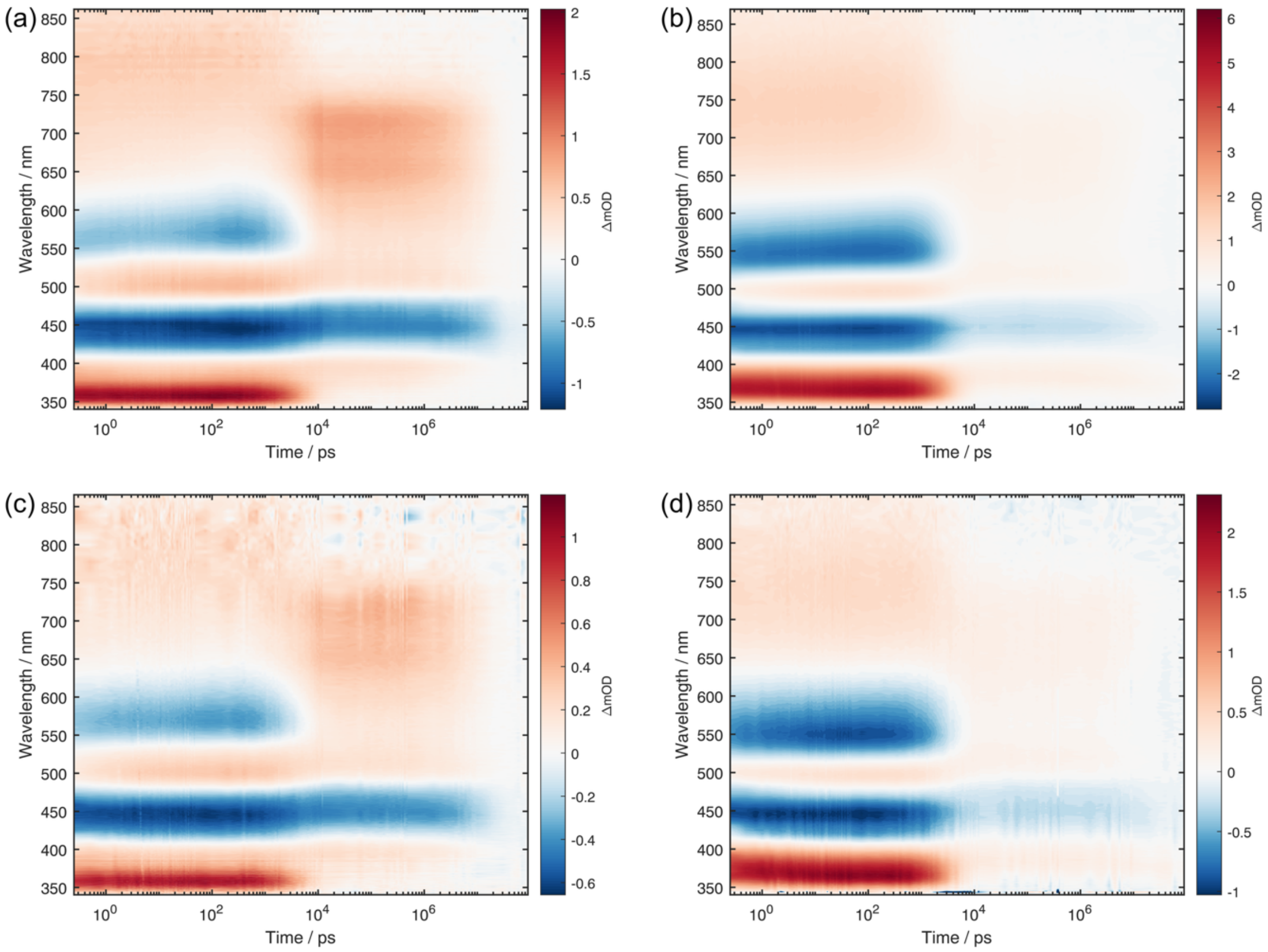

Figure S6. The false-colour contour plots of the riboflavin TA data in water (a,c) and DMSO (b,d). The samples are pumped at (a) 445 nm, (b) 448 nm, (c) 373 nm, and (d) 344 nm, respectively. All data were collected with dissolved oxygen purged.

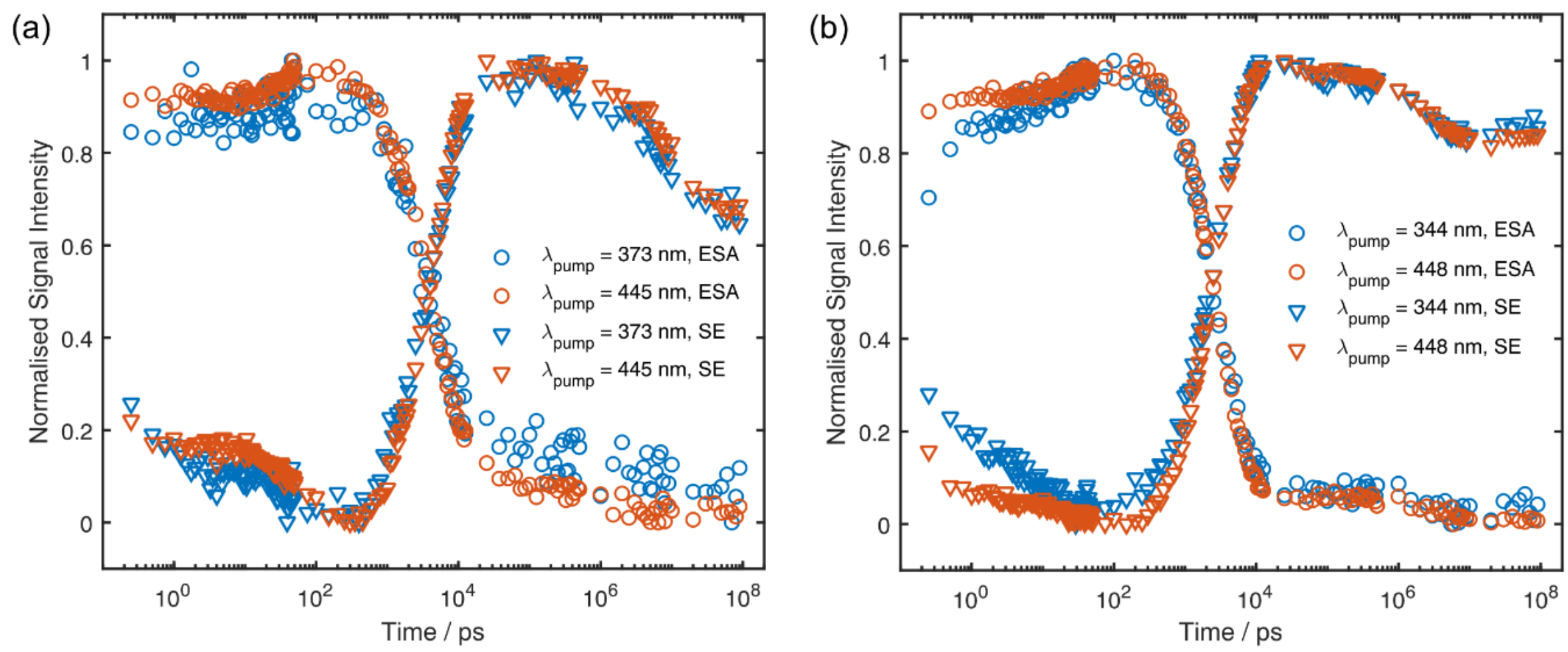

Figure S7. Normalised transient absorption intensity at wavelengths characteristic of ESA and SE of riboflavin in (a) water and (b) DMSO.

S3.3 Effect of Removing Dissolved Oxygen from Solutions on the Transient Dynamics

Data with and without dissolved oxygen present enabled us to confirm the assignment of triplet riboflavin species, as it is readily quenched by O_2. This effect is demonstrated by the kinetic traces for the GSB recovery and triplet decay in Figure S8.

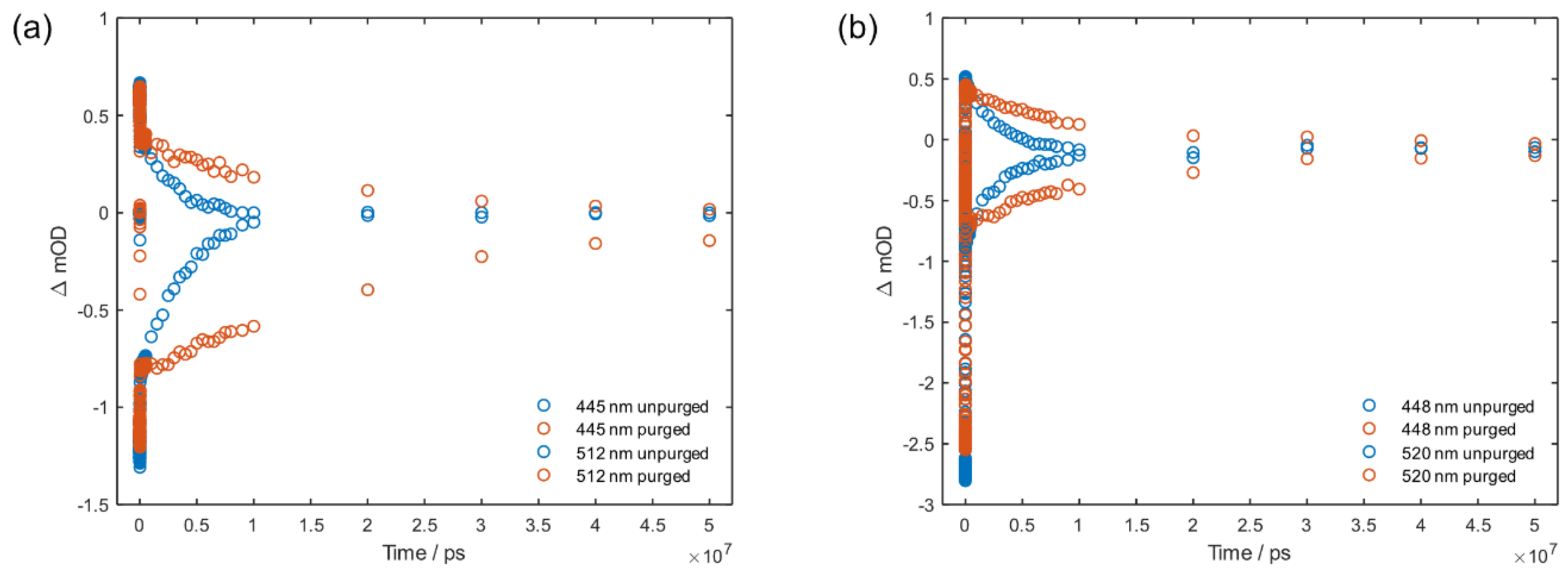

Figure S8. Comparison of longer time dynamics for purged and unpurged RF in (a) water and (b) DMSO.

S3.4 Comparison of Riboflavin in DMSO and DMSO-d_6

Our studies show that the S_1 lifetime of riboflavin in DMSO and DMSO-d_6 varies by 12.5% (2.66 ± 0.03 ns *vs* 3.04 ± 0.07 ns, respectively). Fluorescence quantum yield measurements of riboflavin in DMSO-d_6 compared to DMSO also exhibit a 12.5% decrease (21.0 ± 0.7% and 24.0 ± 2.0 %, respectively). Using eq. S1, the radiative rate constant for riboflavin in both DMSO and DMSO-d_6 are thus determined to be the same (8.0×10^7 s^{-1}). The relative amplitude of the two phases of the bleach recovery in TRIR measurements returns the same triplet quantum yield (0.18) within error. Thus, differences in the S_1 lifetime must originate from a faster rate of internal conversion in DMSO-d_6 compared to DMSO.

This interesting result echoes studies by Maillard *et al.*[37] and Kubitz *et al.*,[38] who observed that the fluorescence lifetime of common organic fluorophores and substituted flavins was altered by changing solvent from H_2O to D_2O. They determined that this difference originated from a resonant energy transfer from the solute S_1 excitation energy to multiple quanta of high-frequency vibrational stretching modes in the solvent *via* a through-space Förster-type mechanism. Solvent deuteration changes the anharmonic ladder of O–H or O–D acceptor modes, detuning them from the S_1 energy level in H_2O *cf.* D_2O, leading to a lower IC rate. Critically, it was determined that this mechanism was not driven through hydrogen bonding.

We speculate that one possible explanation that could rationalise the difference in riboflavin IC rates between DMSO and DMSO-d_6 may be a similar mechanism to that proposed by Maillard *et al.*[37] and Kubitz *et al.*[38]. It would require the C–D or C–H vibrations (anharmonicity constants, $\omega_e x_e$, ~ 30 and 17 cm^{-1} in $(CH_3)_2SO$[39] and $(CD_3)_2SO$,[40] respectively) on the DMSO methyl groups to act as acceptor modes.

S3.5 Global Analysis

A global analysis was also conducted, assuming a linear A → B→ C kinetic scheme, using our home-built MATLAB script. This returned three time constants and the species associated spectra (SAS) for A, B and C.

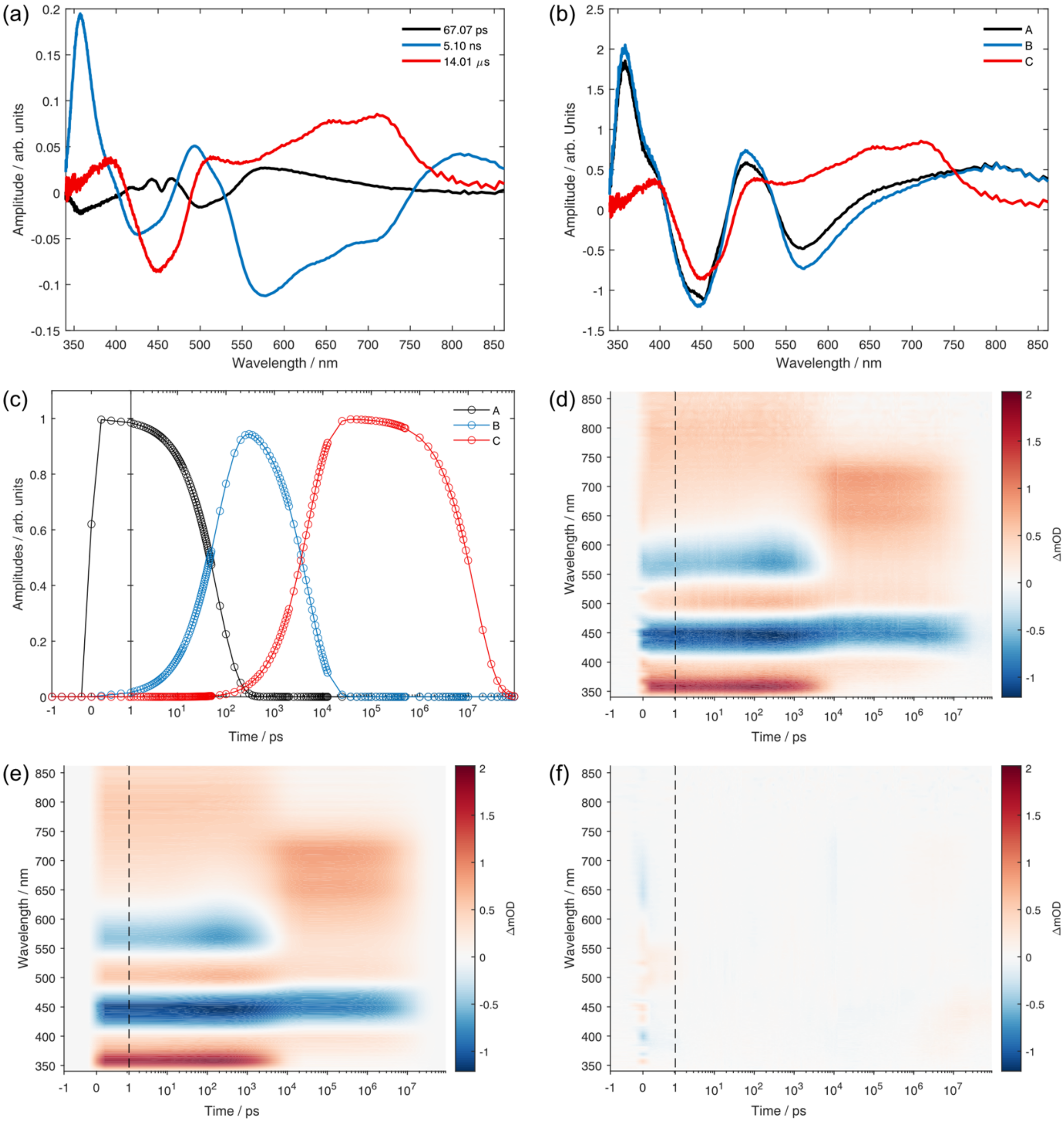

Figure S9. Global analysis results for the water TA data exciting to the lowest excited state. The (a) decay- and (b) species-associated spectra (SAS) are shown with (c) the kinetics associated with the latter. The contour plots for (d) the original data and (e) the one reconstructed from the SAS are shown, with (f) the residuals plots highlighting the discrepancies between the fit and the data.

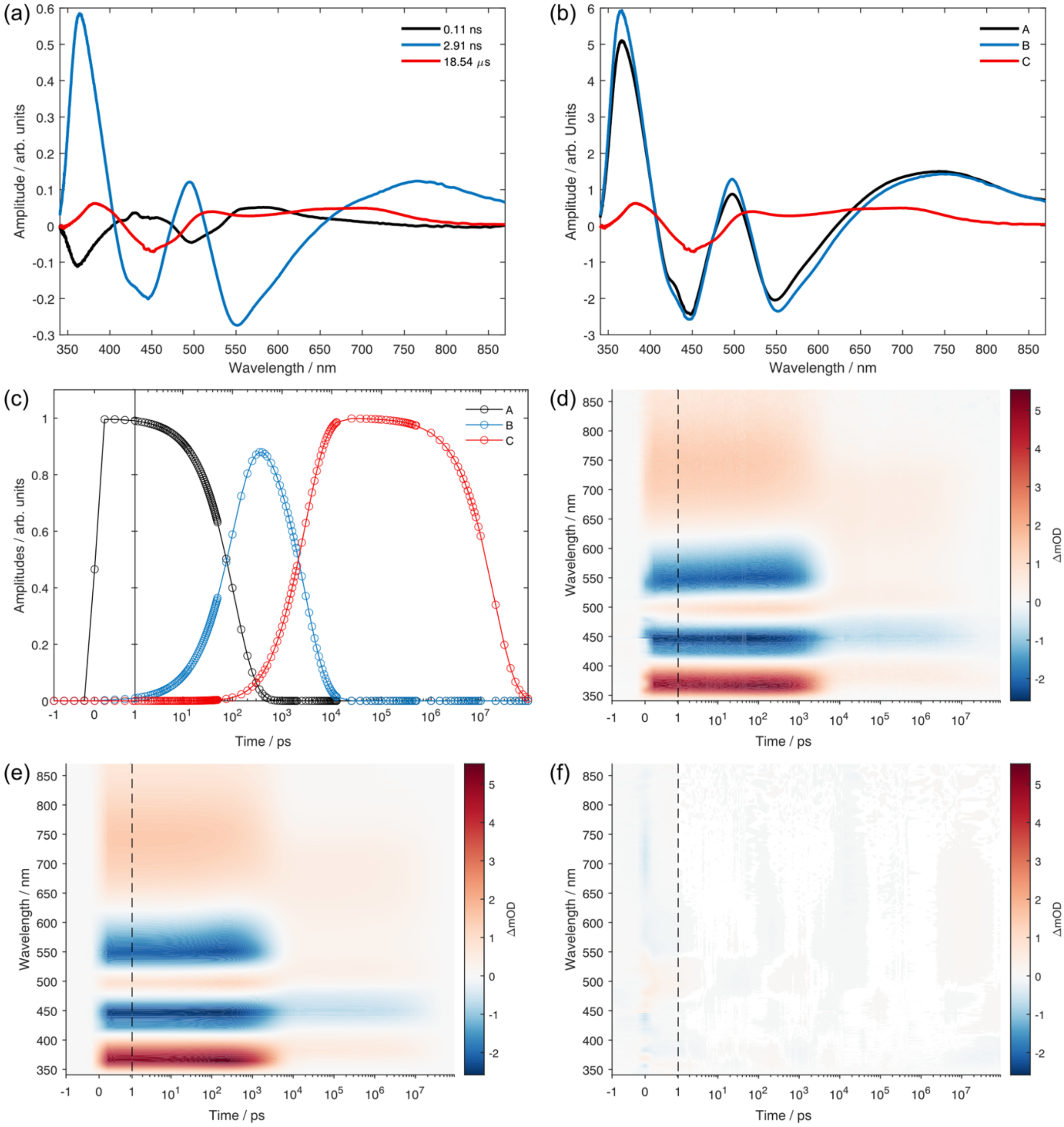

Figure S10. Global analysis results for the DMSO TA data exciting to the lowest excited state. The (a) decay- and (b) SAS are shown with (c) the kinetics associated with the latter. The contour plots for (d) the original data and (e) the one reconstructed from the SAS are shown, with (f) the residuals plots highlighting the discrepancies between the fit and the data.

The species-associated spectra of species B and C correspond well with the spectral signatures assigned to the singlet and triplet riboflavin, respectively, in the main text, and species A is assigned to the hot S_1 state. Combinedly, they manage to reconstruct the TA data with high fidelity, and reproduce the original kinetics. The major discrepancies arise around time zero, where the signal is complex and also contains contributions from the ultrafast response.

S3.6 Time Resolved Infrared Spectroscopy

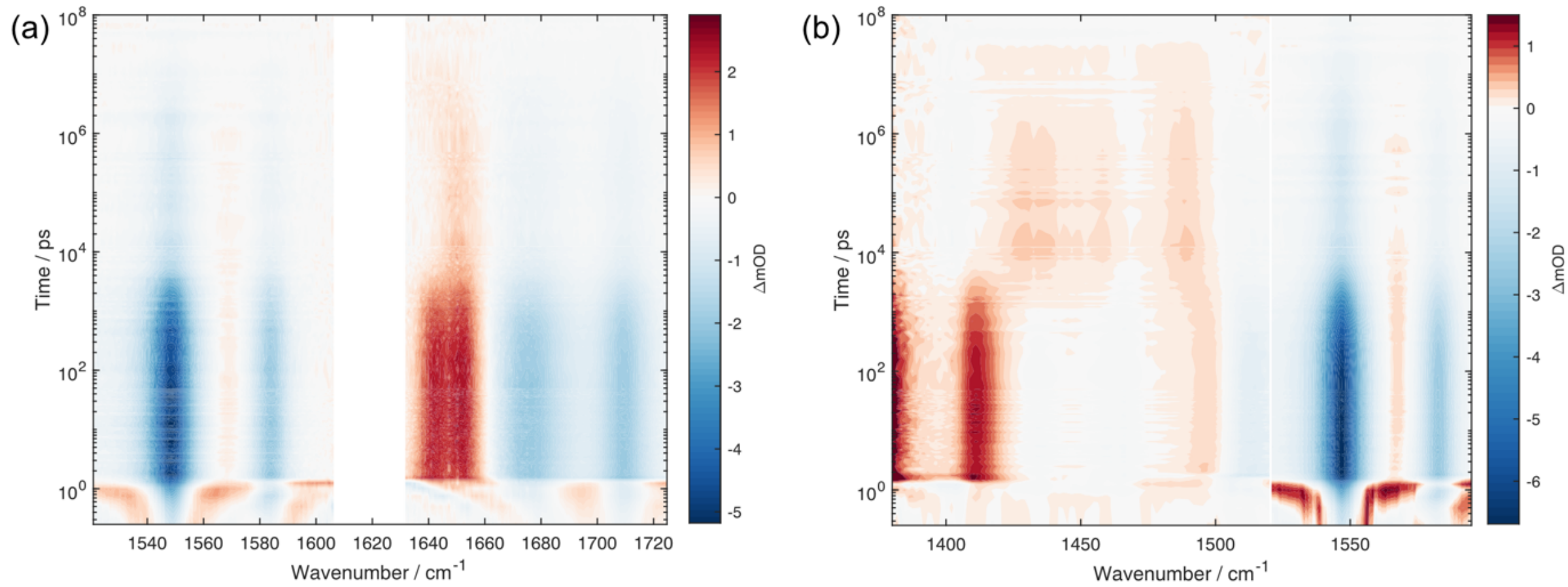

Figure S11. The false-colour contour plots of the full riboflavin TRIR datasets in nitrogen-purged (a) DMSO and (b) DMSO-d_6.

S3.6 Influence of Solvent H-bonding on the Photophysical Properties of Riboflavin

Table S1. [a]Abraham's hydrogen bonding acidity and basicity parameters from ref 41; [b] various experimentally determined and derived photophysical properties of riboflavin determined in the present study; [c] literature values riboflavin photophysical properties, where SB corresponds to values obtained from a Strickler-Berg analysis, from ref. 42; [d] Experimentally derived non-radiative rate constant from this study calculated using eq. S5.

**Solvent**	α_2^H [a]	β_2^H [a]	Φ_F / ns [b]	Φ_F / ns [c]	τ_F / ns [b]	τ_F / ns [c]	k_F / s^{-1} [b]	k_F / s^{-1} [c]	k_F(SB) / s^{-1} [c]	k_{NR} / s^{-1} [d]	k_{NR} / s^{-1} [c]
DMSO	0.00	0.78	0.24 ± 0.02	0.22	3.2 ± 0.1	2.97	$(8.0 \pm 0.7)\times10^7$	4.89×10^7	7.41×10^7	$(2.33 \pm 0.12)\times10^8$	2.60×10^8
Water	0.35	0.38	0.28 ± 0.04	0.26	5.1 ± 0.1	4.5	$(5.6 \pm 0.8)\times10^7$	5.78×10^7	5.85×10^7	$(1.40 \pm 0.09)\times10^8$	1.60×10^8
Methanol	0.37	0.41	—	0.39	—	6.55	—	5.95×10^7	—	—	9.30×10^7
Acetonitrile	0.09	0.44	—	0.24	—	3.84	—	6.25×10^7	—	—	2.00×10^8

S4. Computational Results

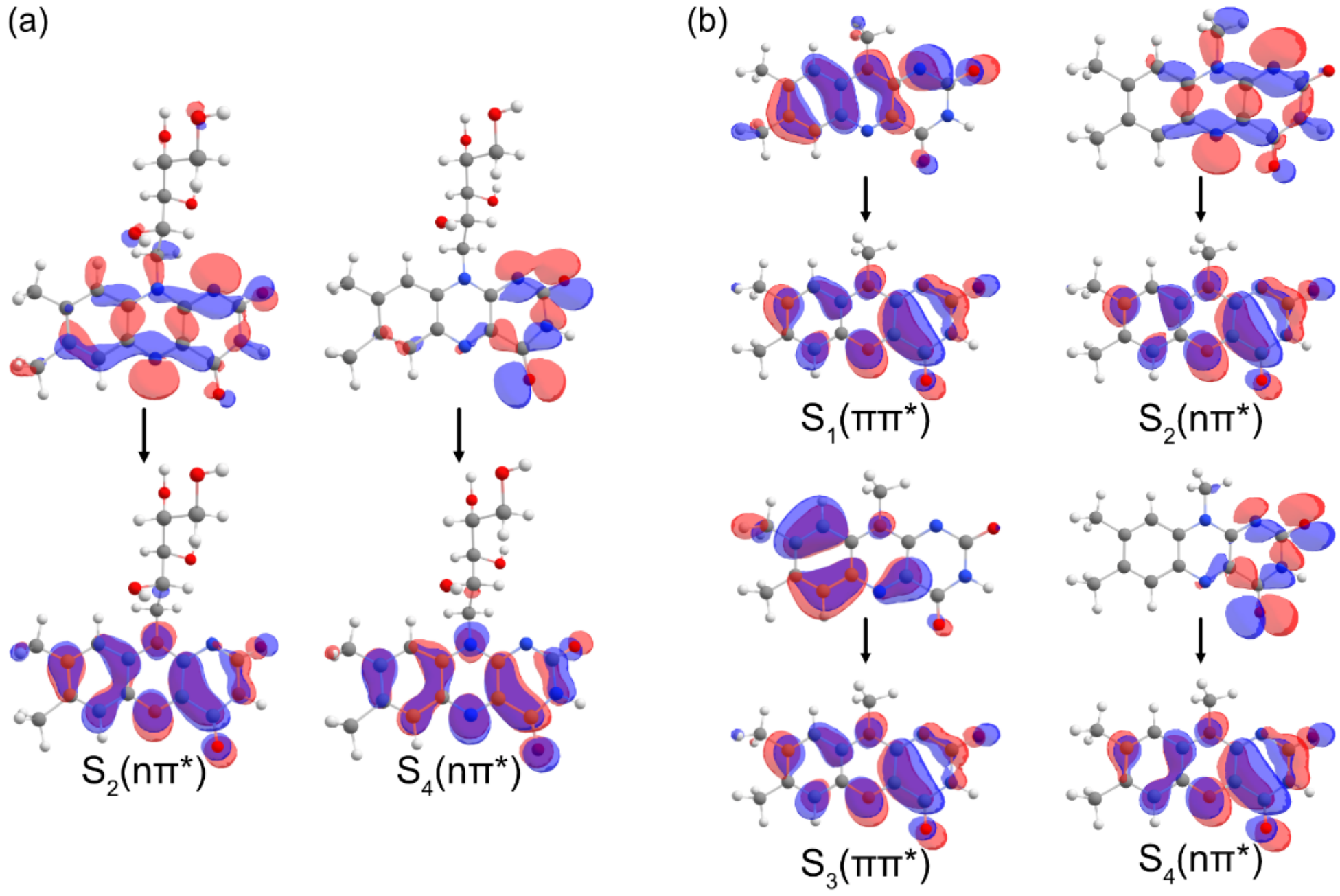

Figure S12. (a) The relevant natural transition orbitals (NTOs) for low-lying nπ* transitions for a sample snapshot of riboflavin from QM/MM**. (b)** NTOs for low-lying *ππ** and nπ* transitions for lumiflavin**,** calculated [29]from a RI-CC2 excitation energy calculation, on the DFT CPCM (water) S_{0min} structure.[30] The arrows connect the hole and particle NTOs associated with each electronic excitation.

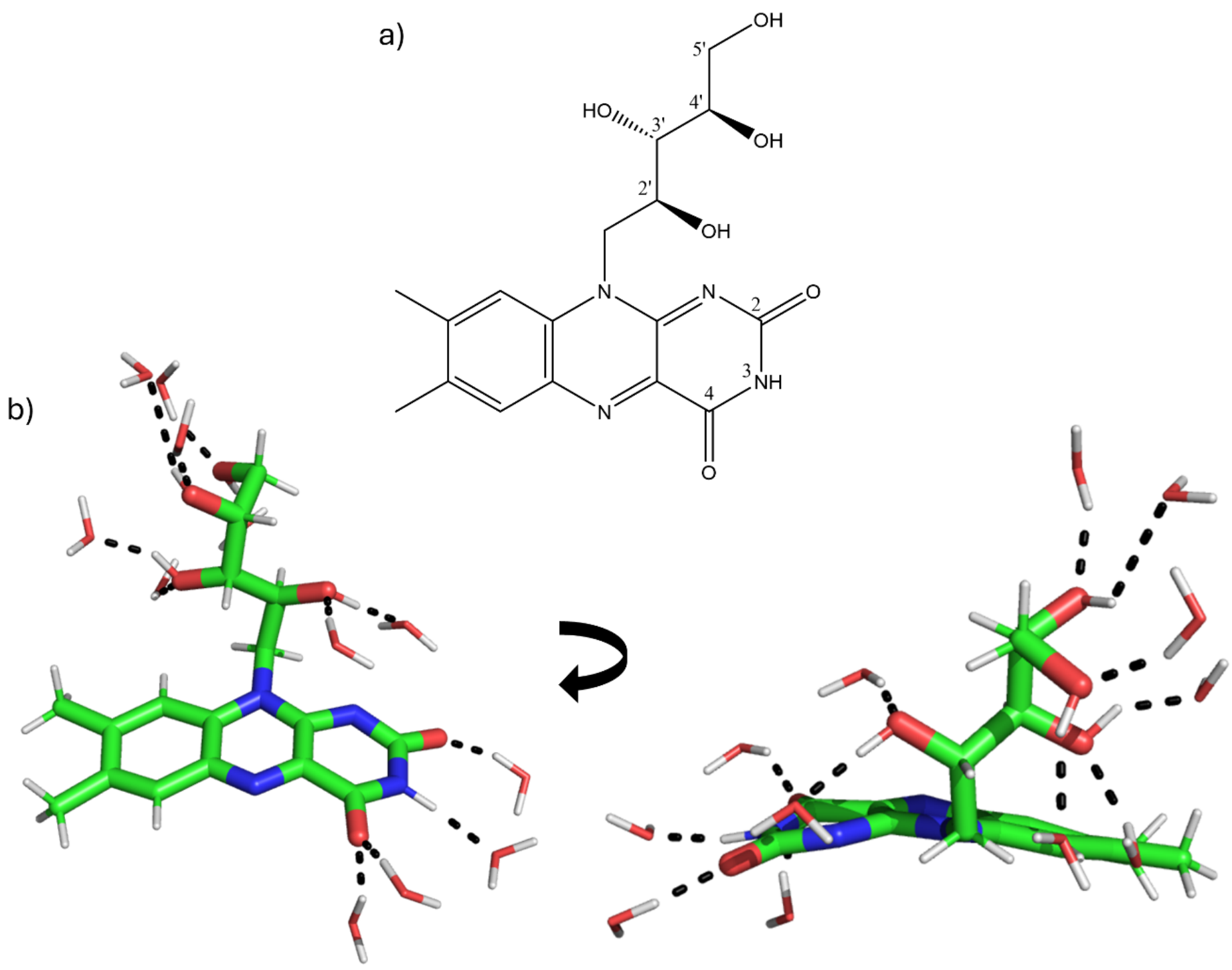

Figure S13. (a) Riboflavin chemical structure, with only carbon atoms attached to functional groups involved in persistent hydrogen bonding labelled (b) Two views of a representative snapshot illustrating persistent hydrogen bonds (represented by dashed lines) between riboflavin and water in QM/MM MD simulations.

Table S2. Analysis of the QM/MM MD simulation for persistent hydrogen bonds formed between riboflavin (RBF) and water molecules as a function of H-bond acceptor and donor (defined in Figure S13), where ISA = isoalloxazine ring, Rb = ribityl chain.

H-Bond Acceptor	H-Bond Donor	Average number of H-Bonds to Riboflavin
RBF O4 (ISA)	Water	1.611
RBF O2 (ISA)	Water	1.053
Water	RBF O3' (Rb)	0.959
Water	RBF O5' (Rb)	0.922
Water	RBF O2' (Rb)	0.898
RBF O3' (Rb)	Water	0.830
RBF O4' (Rb)	Water	0.814
RBF O5' (Rb)	Water	0.777
Water	RBF N3 (ISA)	0.703
RBF O2' (Rb)	Water	0.587

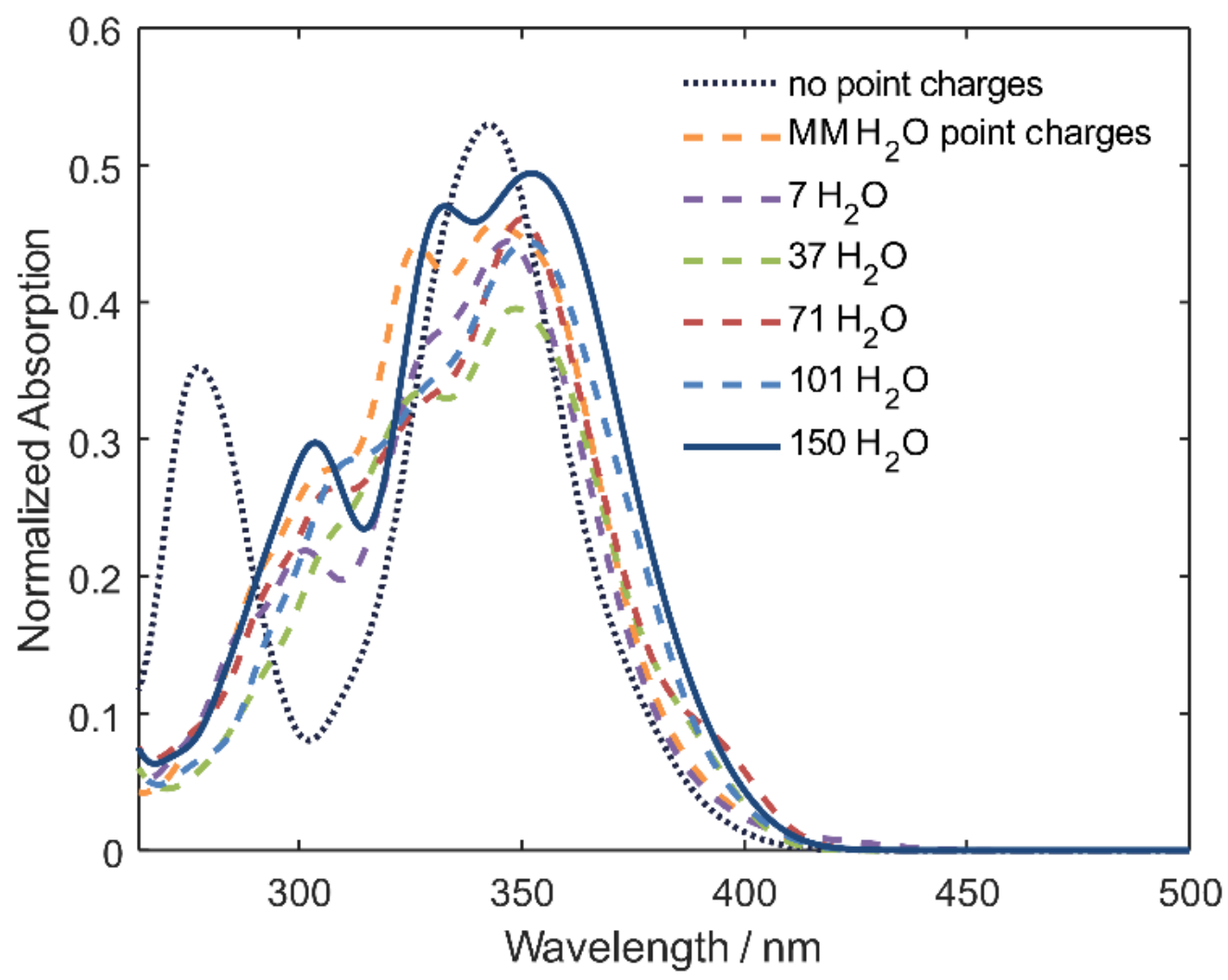

Figure S14. The computed QM/MM spectra for riboflavin. The excitation energies for each snapshot are calculated in vacuum, with MM point charges, and then with a systematic increase of water molecules in the QM region. The 7 sets of calculations are superimposed.

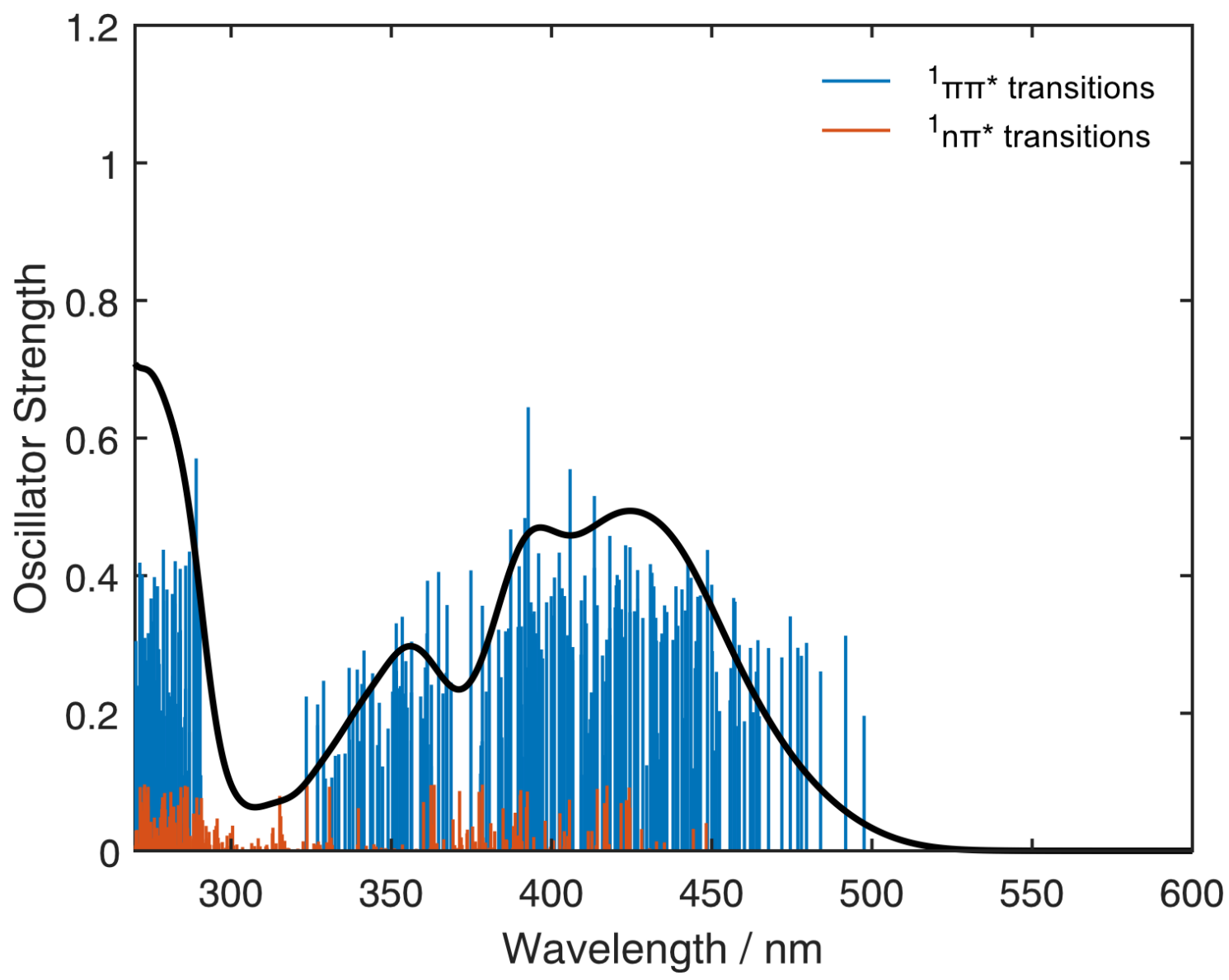

Figure S15. Calculated QM/MM absorption spectrum of riboflavin in water (within a shell of 150 QM water molecules) at the LR-TDDFT/TDA/ωB97X-D3/def2-SVP level of theory. Displayed both as a stick spectrum, corresponding to the individual electronic transitions, and a continuous spectrum broadened with a Gaussian function using a FWHM = 0.165 eV. The broadened continuous spectrum is normalised to λ = 222 nm (outside of shown range), whereas the stick spectra are shown as oscillator strengths. $^1\pi\pi^*$ states are labelled in blue and $^1n\pi^*$ labelled in red. The mean oscillator strength of the $^1n\pi^*$ and $^1\pi\pi^*$ transitions were calculated to be 0.018. and 0.216, respectively.

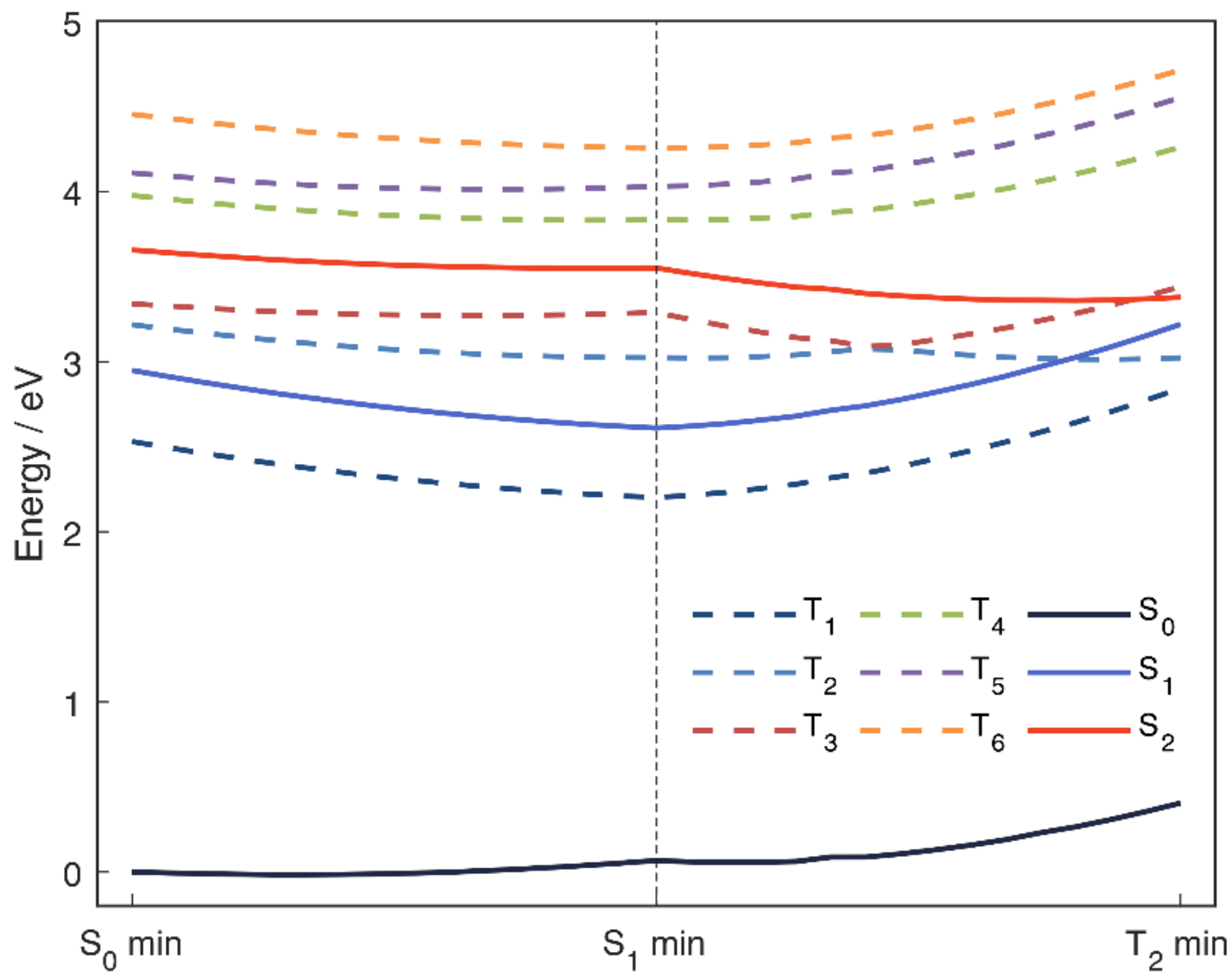

Figure S16. Geodesic path for S_0–S_1 and S_1–T_2 of lumiflavin in DMSO calculated on (LR-TD)DFT structures using RI-CC2 for the excitation energies, where the S_1 and T_2 minima have ππ* and *n*π* character.

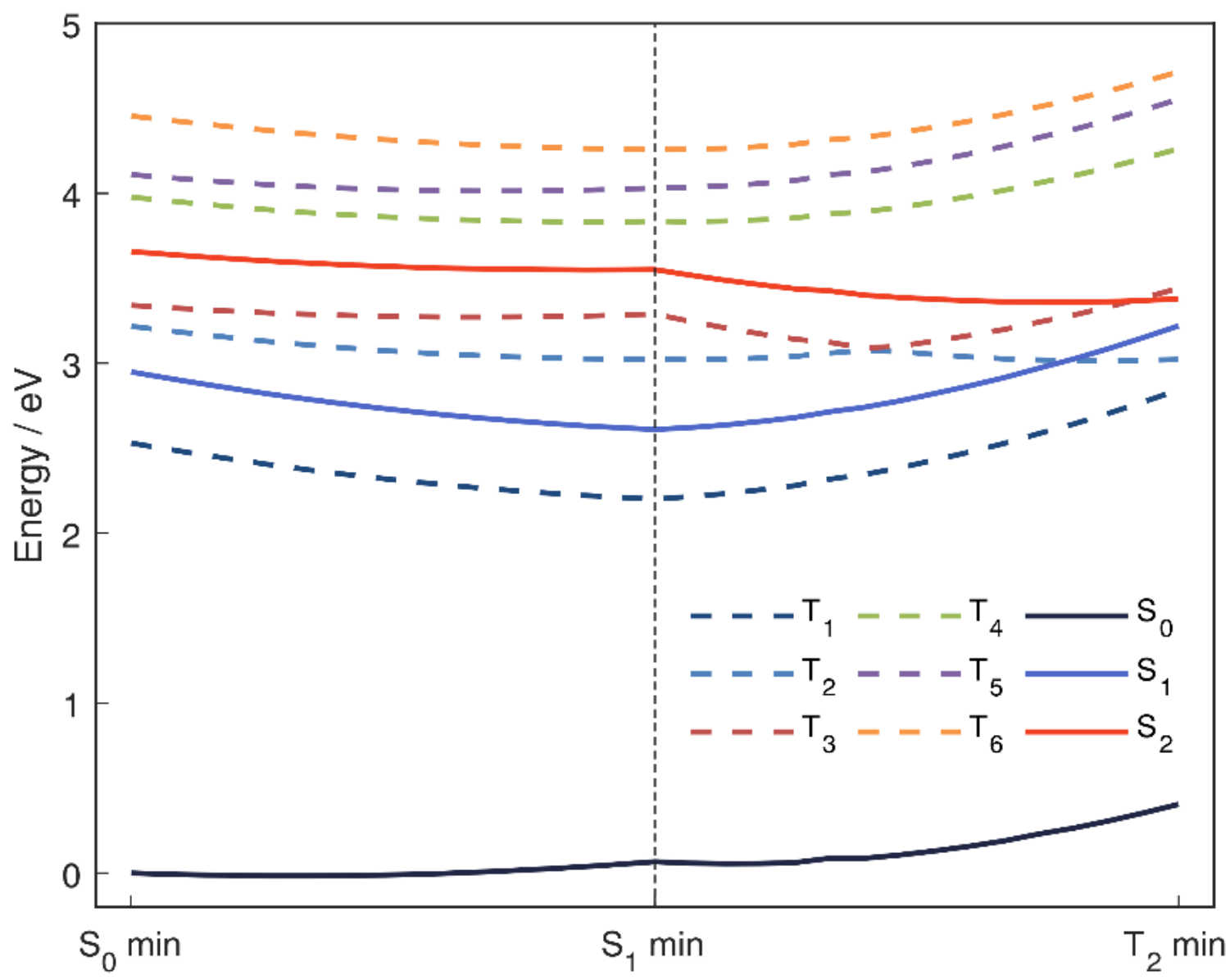

Figure S17. Geodesic path for S_0–S_1 and S_1–T_2 of lumiflavin in water calculated on (LR-TD)DFT structures using RI-CC2 for the excitation energies, where the S_1 and T_2 minima have ππ* and *n*π* character.

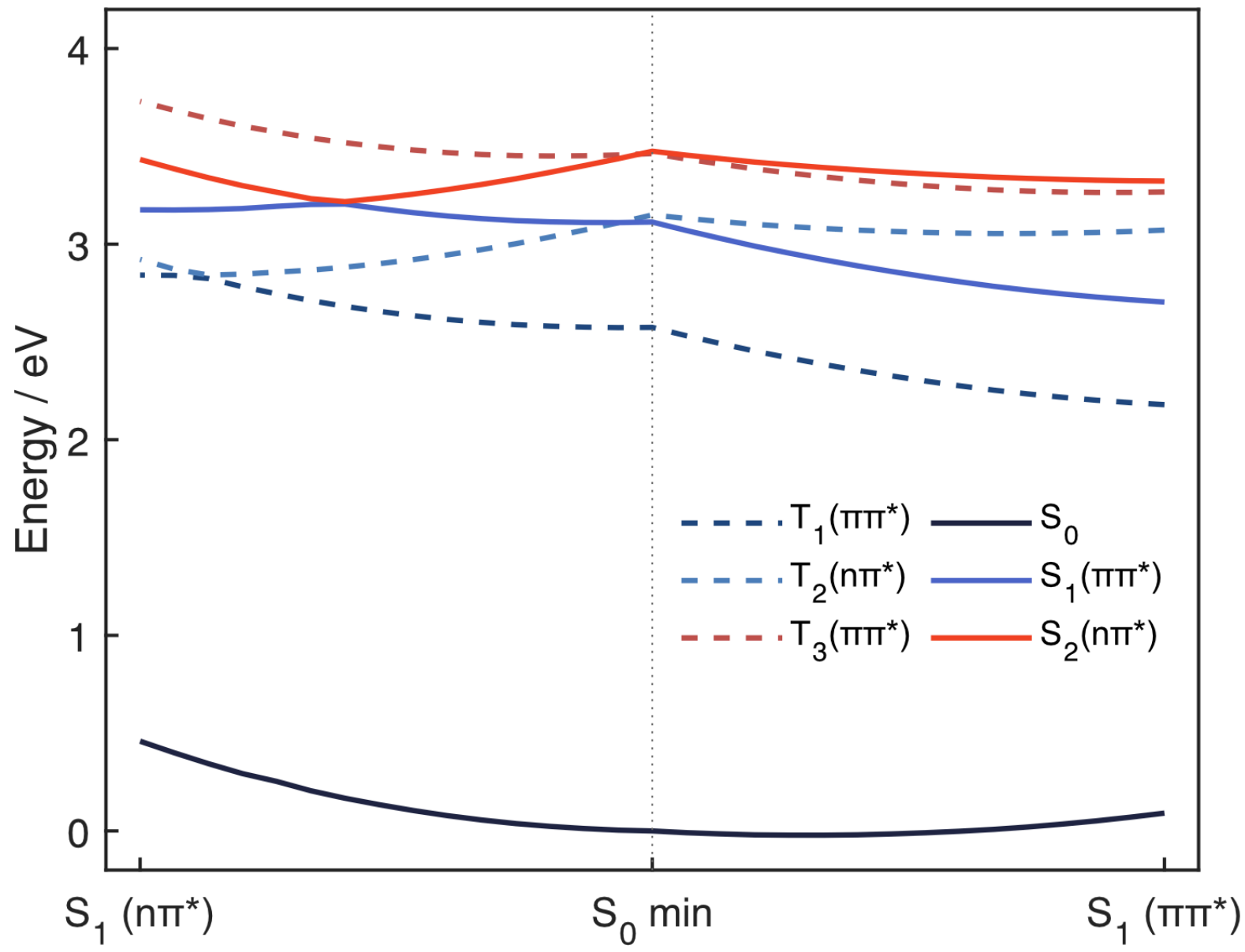

Figure S18. Geodesic path for (left) S_0–S_1 ($n\pi^*$) and (right) S_0–S_1 ($\pi\pi^*$) states of riboflavin in the gas phase, calculated based on (LR-TD)DFT structures using RI-CC2 for the excitation energies. We note that the electronic character of T_1 and T_2 swap when approaching the S_1 ($n\pi^*$) geometry.

References

(1) Feng, Y.; Vinogradov, I.; Ge, N.-H. General noise suppression scheme with reference detection in heterodyne nonlinear spectroscopy. *Optics Express* **2017**, *25* (21), 26262-26279.
(2) Feng, Y.; Vinogradov, I.; Ge, N.-H. Optimized noise reduction scheme for heterodyne spectroscopy using array detectors. *Optics Express* **2019**, *27* (15), 20323-20346.
(3) Bressan, G.; Heisler, I. A.; Greetham, G. M.; Edmeades, A.; Meech, S. R. Half-broadband two-dimensional electronic spectroscopy with active noise reduction. *Optics Express* **2023**, *31* (25), 42687-42700.
(4) Farrell, K. M.; Ostrander, J. S.; Jones, A. C.; Yakami, B. R.; Dicke, S. S.; Middleton, C. T.; Hamm, P.; Zanni, M. T. Shot-to-shot 2D IR spectroscopy at 100 kHz using a Yb laser and custom-designed electronics. *Optics Express* **2020**, *28* (22), 33584-33602.
(5) Kübel, J.; Westenhoff, S.; Maj, M. Giving voice to the weak: Application of active noise reduction in transient infrared spectroscopy. *Chemical Physics Letters* **2021**, *783*, 139059.
(6) Megerle, U.; Pugliesi, I.; Schriever, C.; Sailer, C. F.; Riedle, E. Sub-50 fs broadband absorption spectroscopy with tunable excitation: putting the analysis of ultrafast molecular dynamics on solid ground. *Applied Physics B* **2009**, *96* (2), 215-231.
(7) Dobryakov, A. L.; Kovalenko, S. A.; Weigel, A.; Pérez-Lustres, J. L.; Lange, J.; Müller, A.; Ernsting, N. P. Femtosecond pump/supercontinuum-probe spectroscopy: Optimized setup and signal analysis for single-shot spectral referencing. *Review of Scientific Instruments* **2010**, *81* (11), 113106.
(8) Chai, J.-D.; Head-Gordon, M. Long-range corrected hybrid density functionals with damped atom–atom dispersion corrections. *Physical Chemistry Chemical Physics* **2008**, *10* (44), 6615-6620.
(9) Rappoport, D.; Furche, F. Property-optimized Gaussian basis sets for molecular response calculations. *The Journal of Chemical Physics* **2010**, *133* (13), 134105.
(10) Ufimtsev, I. S.; Martínez, T. J. Quantum Chemistry on Graphical Processing Units. 1. Strategies for Two-Electron Integral Evaluation. *Journal of Chemical Theory and Computation* **2008**, *4* (2), 222-231.
(11) Ufimtsev, I. S.; Martinez, T. J. Quantum Chemistry on Graphical Processing Units. 2. Direct Self-Consistent-Field Implementation. *Journal of Chemical Theory and Computation* **2009**, *5* (4), 1004-1015.
(12) Ufimtsev, I. S.; Martinez, T. J. Quantum Chemistry on Graphical Processing Units. 3. Analytical Energy Gradients, Geometry Optimization, and First Principles Molecular Dynamics. *Journal of Chemical Theory and Computation* **2009**, *5* (10), 2619-2628.
(13) Case, D. A.; Cerutti, D. S.; Cruzeiro, V. W. D.; Darden, T. A.; Duke, R. E.; Ghazimirsaeed, M.; Giambaşu, G. M.; Giese, T. J.; Götz, A. W.; Harris, J. A.; Kasavajhala, K.; Lee, T.-S.; Li, Z.; Lin, C.; Liu, J.; Miao, Y.; Salomon-Ferrrer, R.; Shen, J.; Snyder, R.; Swails, J.; Walker, R. C.; Wang, J.; Wu, X.; Zeng, J.; Cheatham Iii, T. E.; Roe, D. R.; Roitberg, A.; Simmerling, C.; York, D. M.; Nagan, M. C.; Merz, K. M., Jr. Recent Developments in Amber Biomolecular Simulations. *Journal of Chemical Information and Modeling* **2025**, *65* (15), 7835-7843.
(14) Case, D. A.; Aktulga, H. M.; Belfon, K.; Cerutti, D. S.; Cisneros, G. A.; Cruzeiro, V. W. D.; Forouzesh, N.; Giese, T. J.; Götz, A. W.; Gohlke, H.; Izadi, S.; Kasavajhala, K.; Kaymak, M. C.; King, E.; Kurtzman, T.; Lee, T.-S.; Li, P.; Liu, J.; Luchko, T.; Luo, R.; Manathunga, M.; Machado, M. R.; Nguyen, H. M.; O'Hearn, K. A.; Onufriev, A. V.; Pan, F.; Pantano, S.; Qi, R.; Rahnamoun, A.; Risheh, A.; Schott-Verdugo, S.; Shajan, A.; Swails, J.; Wang, J.; Wei, H.; Wu, X.; Wu, Y.; Zhang, S.; Zhao, S.; Zhu, Q.; Cheatham, T. E., III; Roe, D. R.; Roitberg, A.; Simmerling, C.; York, D. M.; Nagan, M. C.; Merz, K. M., Jr. AmberTools. *Journal of Chemical Information and Modeling* **2023**, *63* (20), 6183-6191.

(15) Ryckaert, J.-P.; Ciccotti, G.; Berendsen, H. J. C. Numerical integration of the cartesian equations of motion of a system with constraints: molecular dynamics of n-alkanes. *Journal of Computational Physics* **1977**, *23* (3), 327-341.
(16) Darden, T.; York, D.; Pedersen, L. Particle mesh Ewald: An N·log(N) method for Ewald sums in large systems. *The Journal of Chemical Physics* **1993**, *98* (12), 10089-10092.
(17) Davidchack, R. L.; Handel, R.; Tretyakov, M. V. Langevin thermostat for rigid body dynamics. *The Journal of Chemical Physics* **2009**, *130* (23), 234101.
(18) Berendsen, H. J. C.; Postma, J. P. M.; van Gunsteren, W. F.; DiNola, A.; Haak, J. R. Molecular dynamics with coupling to an external bath. *The Journal of Chemical Physics* **1984**, *81* (8), 3684-3690.
(19) Debiec, K. T.; Cerutti, D. S.; Baker, L. R.; Gronenborn, A. M.; Case, D. A.; Chong, L. T. Further along the Road Less Traveled: AMBER ff15ipq, an Original Protein Force Field Built on a Self-Consistent Physical Model. *Journal of Chemical Theory and Computation* **2016**, *12* (8), 3926-3947.
(20) Cerutti, D. S.; Rice, J. E.; Swope, W. C.; Case, D. A. Derivation of Fixed Partial Charges for Amino Acids Accommodating a Specific Water Model and Implicit Polarization. *The Journal of Physical Chemistry B* **2013**, *117* (8), 2328-2338.
(21) Cerutti, D. S.; Swope, W. C.; Rice, J. E.; Case, D. A. ff14ipq: A Self-Consistent Force Field for Condensed-Phase Simulations of Proteins. *Journal of Chemical Theory and Computation* **2014**, *10* (10), 4515-4534.
(22) Rao, A. G.; Wiebeler, C.; Sen, S.; Cerutti, D. S.; Schapiro, I. Histidine protonation controls structural heterogeneity in the cyanobacteriochrome AnPixJg2. *Physical Chemistry Chemical Physics* **2021**, *23* (12), 7359-7367.
(23) Neese, F.; Wennmohs, F.; Becker, U.; Riplinger, C. The ORCA quantum chemistry program package. *The Journal of Chemical Physics* **2020**, *152* (22), 224108.
(24) Cieplak, P.; Cornell, W. D.; Bayly, C.; Kollman, P. A. Application of the multimolecule and multiconformational RESP methodology to biopolymers: Charge derivation for DNA, RNA, and proteins. *Journal of Computational Chemistry* **1995**, *16* (11), 1357-1377.
(25) Cruzeiro, V. W. D.; Wang, Y.; Pieri, E.; Hohenstein, E. G.; Martínez, T. J. TeraChem protocol buffers (TCPB): Accelerating QM and QM/MM simulations with a client–server model. *The Journal of Chemical Physics* **2023**, *158* (4), 044801.
(26) Jorgensen, W. L.; Chandrasekhar, J.; Madura, J. D.; Impey, R. W.; Klein, M. L. Comparison of simple potential functions for simulating liquid water. *The Journal of Chemical Physics* **1983**, *79* (2), 926-935.
(27) Hirata, S.; Head-Gordon, M. Time-dependent density functional theory within the Tamm–Dancoff approximation. *Chemical Physics Letters* **1999**, *314* (3), 291-299.
(28) Hollas, D.; Curchod, B. F. E. AtmoSpec–A Tool to Calculate Photoabsorption Cross-Sections for Atmospheric Volatile Organic Compounds. *The Journal of Physical Chemistry A* **2024**, *128* (39), 8580-8590.
(29) Plasser, F. TheoDORE: A toolbox for a detailed and automated analysis of electronic excited state computations. *The Journal of Chemical Physics* **2020**, *152* (8), 084108.
(30) *Chemcraft - graphical software for visualization of quantum chemistry computations.* https://www.chemcraftprog.com.
(31) Zhu, X.; Thompson, K. C.; Martínez, T. J. Geodesic interpolation for reaction pathways. *The Journal of Chemical Physics* **2019**, *150* (16), 164103
(32) Christiansen, O.; Koch, H.; Jørgensen, P. The second-order approximate coupled cluster singles and doubles model CC2. *Chemical Physics Letters* **1995**, *243* (5), 409-418.
(33) Balasubramani, S. G.; Chen, G. P.; Coriani, S.; Diedenhofen, M.; Frank, M. S.; Franzke, Y. J.; Furche, F.; Grotjahn, R.; Harding, M. E.; Hättig, C.; Hellweg, A.; Helmich-Paris, B.; Holzer, C.; Huniar, U.; Kaupp, M.; Marefat Khah, A.; Karbalaei Khani, S.; Müller, T.; Mack,

F.; Nguyen, B. D.; Parker, S. M.; Perlt, E.; Rappoport, D.; Reiter, K.; Roy, S.; Rückert, M.; Schmitz, G.; Sierka, M.; Tapavicza, E.; Tew, D. P.; van Wüllen, C.; Voora, V. K.; Weigend, F.; Wodyński, A.; Yu, J. M. TURBOMOLE: Modular program suite for ab initio quantum-chemical and condensed-matter simulations. *The Journal of Chemical Physics* **2020**, *152* (18), 184107.
(34) Schäfer, A.; Klamt, A.; Sattel, D.; Lohrenz, J. C. W.; Eckert, F. COSMO Implementation in TURBOMOLE: Extension of an efficient quantum chemical code towards liquid systems. *Physical Chemistry Chemical Physics* **2000**, *2* (10), 2187-2193.
(35) Cerezo, J.; Santoro, F. FCclasses3: Vibrationally-resolved spectra simulated at the edge of the harmonic approximation. *Journal of Computational Chemistry* **2023**, *44* (4), 626-643.
(36) Peach, M. J. G.; Williamson, M. J.; Tozer, D. J. Influence of Triplet Instabilities in TDDFT. *Journal of Chemical Theory and Computation* **2011**, *7* (11), 3578-3585.
(37) Maillard, J.; Klehs, K.; Rumble, C.; Vauthey, E.; Heilemann, M.; Fürstenberg, A. Universal quenching of common fluorescent probes by water and alcohols. *Chemical Science* **2021**, *12* (4), 1352-1362.
(38) Kubitz, M. K.; Haselbach, W.; Sretenović, D.; Bracker, M.; Kleinschmidt, M.; Kühnemuth, R.; Seidel, C. A. M.; Gilch, P.; Czekelius, C. Increasing the Fluorescence Quantum Yield and Lifetime of the Flavin Chromophore by Rational Design. *ChemPhotoChem* **2023**, *7* (7), e202200334.
(39) Wallace, V. M.; Dhumal, N. R.; Zehentbauer, F. M.; Kim, H. J.; Kiefer, J. Revisiting the Aqueous Solutions of Dimethyl Sulfoxide by Spectroscopy in the Mid- and Near-Infrared: Experiments and Car–Parrinello Simulations. *The Journal of Physical Chemistry B* **2015**, *119* (46), 14780-14789.
(40) Pruccoli, A.; Kocademir, M.; Winterhalder, M. J.; Zumbusch, A. Electronically Preresonant Stimulated Raman Scattering Microscopy of Weakly Fluorescing Chromophores. *The Journal of Physical Chemistry B* **2023**, *127* (27), 6029-6037.
(41) Abraham, M. H. Scales of solute hydrogen-bonding: their construction and application to physicochemical and biochemical processes. *Chemical Society Reviews* **1993**, *22* (2), 73-83.
(42) Zirak, P.; Penzkofer, A.; Mathes, T.; Hegemann, P. Photo-dynamics of roseoflavin and riboflavin in aqueous and organic solvents. *Chemical Physics* **2009**, *358* (1), 111-122.
